# Explainable machine learning for predicting disease flares in axial spondyloarthritis: A real-world electronic health record-based pilot study

**DOI:** 10.1177/20552076261433513

**Published:** 2026-04-25

**Authors:** Antoni Chan, Pradip Moon, Weizi Li, Eghosa Bazuaye

**Affiliations:** 1 Informatics Research Centre, 6816 University of Reading, Reading, UK; 2 University Department of Rheumatology, Royal Berkshire NHS Foundation Trust, Reading, UK; 3 Department of Digital Operations, 6818 Royal Berkshire NHS Foundation Trust, Reading, UK

**Keywords:** Axial spondyloarthritis, flare prediction, machine learning, electronic health records, digital health, explainable AI

## Abstract

**Objective:**

Flares of axial spondyloarthritis (axSpA) are common yet unpredictable. We aimed to develop and internally validate a machine learning (ML) model to forecast flares 3, 6, 9 and 12 months ahead using routinely collected electronic health record (EHR) data.

**Methods:**

We performed a retrospective cohort study of 282 axSpA patients (January 2018 to May 2024) in our centre. Flares were defined as a ≥ 2.1 unit rise in Bath Ankylosing Spondylitis Disease Activity Index (BASDAI), ≥ 0.9 unit rise in axSpA Disease Activity Score and clinician confirmed flare. Ninety-eight candidate predictors spanning demographics, patient-reported outcomes, laboratory indices and comorbidities were aggregated into time-series windows. We applied Light Gradient-Boosting Machine (LGBM) and eXtreme Gradient Boosting to forecast the risk of flare. Model interpretability was assessed with SHapley Additive exPlanations (SHAP).

**Results:**

Of 282 patients, 100 (35.5%) experienced at least one flare. Our LGBM model demonstrated key 3-month metrics of: accuracy 0.846 (95% CI 0.545–0.980), sensitivity 0.833 (95% CI 0.358–0.995), specificity 0.857 (95% CI 0.421–0.996) and area under the receiver operating characteristic curve (AUROC) 0.845 (95% CI 0.615–1.000). Performance decreased modestly at 12 months, AUROC 0.773 (95% CI 0.562–0.984). Top SHAP contributors included comorbidity burden, BASDAI, CRP deviation, lymphocyte count, age and deprivation index. Individual-level SHAP plots enabled personalised risk profiles.

**Conclusion:**

This proof-of-concept study demonstrates the feasibility of explainable ML models to predict axSpA flares up to 1 year in advance using real-world EHR data. Embedding the algorithm in electronic records could triage high-risk patients to earlier review and therapy adjustment. This approach offers a novel strategy to inform treat-to-target care pathways and support future integration into digital rheumatology systems.

## Introduction

Axial spondyloarthritis (axSpA) is an immune-mediated condition that affects around 1% of the population and causes inflammation in the spine including the sacroiliac joints and peripheral joints.^1^ Flare of the disease with worsening of the clinical condition can occur in axSpA. This can cause significant issues for the patient including loss of function, mobility and work productivity. The definition of a flare may be from a clinician confirmed encounter or based on the change in the outcome scores such as the Bath Ankylosing Spondylitis Disease Activity Index (BASDAI) or the Axial Spondyloarthritis Disease Activity Score (ASDAS).^2^ ML models have been explored to predict flares such as using wearable activity trackers to monitor physical activity. A reduction in physical activity was used to predict flares.^3^ ML models have also predicted diagnosis and radiographic progression in axSpA.^4,5^ However, there yet no study that have looked at future flare prediction using structured electronic record data. Compared to previous studies, our study sought to develop and internally validate an interpretable ML model that uses routinely collected electronic health record (EHR) data to forecast flares 3, 6, 9 and 12 months ahead. Our overarching hypothesis was that integrating multimodal EHR data including demographics, comorbidities, laboratory results and patient-reported outcomes would enable more accurate flare prediction than single-parameter threshold-based approaches alone.

## Methods

We aimed to develop an interpretable machine learning (ML) model capable of predicting axSpA flares up to 12 months in advance using routinely collected EHR data.

### Patients

Data collection for this study took place within the University Department of Rheumatology at the Royal Berkshire NHS Foundation Trust (UK). The research dataset encompassed 282 axSpA patients from January 2018 until May 2024. Patients were diagnosed before January 2018 and were followed through until May 2024. All axSpA patients fulfilled the ASAS classification criteria.^6^ We excluded patients with < 2 outpatient visits in the study window, incomplete laboratory data at baseline or participation in blinded interventional trials during follow-up.

In total, 282 patients with axSpA (5498 visits) were initially identified from the EHR. After applying data completeness criteria, 262 patients had valid visit and appointment timestamps as well as defined flare or non-flare outcome. For model development, we further restricted the cohort to patients with at least two outpatient visits in the study window, yielding an analysis cohort of 248 patients contributing 5464 visits. Thus, 34 patients (12.1%) were excluded because of insufficient longitudinal or outcome data.

### Ethics and approval

Institutional approval was granted for the study, while patients consented to have their data analysed. The study tracked each patient and assigned categories according to their flare occurrences throughout the study. To maintain privacy and confidentiality, all patient data were de-identified in accordance with ethical standards and UK General Data Protection Regulation guidelines. This study has obtained ethics approval from the Health Research Authority in the UK with reference number IRAS 334608.

### Dependant variable

Our primary outcome measure focused on the presence of flares or non-flare throughout the study period. The variable represented patients’ flare status determined through clinical assessments documented in the electronic patient record. The binary dependent variable served as a critical element for model training to distinguish between flare and non-flare states.

The prediction target in this study was defined at the patient level. For each patient, the binary outcome variable (Appointment Resource) indicated whether they experienced at least one clinically validated flare during the study period. A flare episode was assigned when the following were met: (1) BASDAI increase ≥ 2.1 units^7^ from preceding visit and/or ASDAS increase ≥ 0.9 units; (2) confirmed flare and documentation by a Consultant Rheumatologist in person in clinic. All flare episodes were retrospectively validated by the study team through structured chart review to minimise misclassification.

Because the model was designed to forecast the risk of a future flare at upcoming clinical appointments, flare status was not assigned per-visit or per-interval. Instead, each patient received a single flare/non-flare label, and their longitudinal data across multiple visits were aggregated into fixed-length temporal sequences (13 time points for Light Gradient-Boosting Machine (LGBM) and 14 for eXtreme Gradient Boosting (XGBoost)). This ensured uniform model inputs while maintaining temporal ordering of clinical measurements.

### Horizon construction (3,6,9, and 12 months)

Prediction horizons were used only during model evaluation, and not to create separate flare targets. For each horizon (3,6,9 and 12 months), only patients with at least one recorded clinical observation in the interval were included in evaluation. This approach simulates a realistic clinical scenario in which the model is applied horizon months prior to a scheduled clinic review, using only data available at that time.

Across the full cohort (n = 282), 100 patients (35.5%) experienced ≥ 1 flare and 182 (64.5%) did not. The number of patients contributing to each prediction horizon – each represented once per horizon was:

- 3-month horizon: 54 patients (21 flare, 33 non-flare)
- 6-month horizon: 79 patients (34 flare, 45 non-flare)
- 9-month horizon: 93 patients (39 flare, 54 non-flare)
- 12-month horizon: 100 patients (40 flare, 60 non-flare)

The dependent variable was defined accurately and comprehensively by analysing detailed clinical patient data to identify flares. Equally, the non-flare group was defined as not having the increase in the BASDAI or ASDAS CRP as defined above and confirmed by a Consultant Rheumatologist in person as not having a flare.

### Predictors

The dataset contained multiple categories of information about the patients. This included 98 independent variables across six main categories: (1) demographic features (age, gender, ethnicity, marital status, deprivation index), (2) disease activity and functional scores (BASDAI, ASDAS, BASFI, BASG, Numerical Rating Score (NRS) pain scores), (3) comorbidity indicators, (4) routine blood test results (including haematological, biochemical, and autoimmune markers), (5) derived lab metrics such as deviation from normal reference ranges and (6) physiological measures including height, weight and BMI. The combined dataset having 98 independent variables and detailed information provided in supplementary material section S4 (Table S1). Combining multiple data sources enabled complete patient health assessments, which helped build the predictive model. The patient data were deidentified to ensure privacy and confidentiality, following ethical guidelines and regulatory requirements.

### Data preprocessing

The clinical data required several preprocessing steps before it could be used for flare prediction modelling. The dataset contained structured and semi-structured information arranged in a longitudinal format. The preprocessing pipeline included the following components:

- Temporal sequence construction
  Clinical visit timestamps were used to preserve the temporal structure of each patient's record. We created fixed-length sequences (13 time points for LGBM, 14 for XGBoost) to ensure uniform model inputs. When fewer visits were available, zero-padding was applied at the start of the sequence. The design maintained the chronological sequence of clinical data, which facilitated subsequent sequence-based analysis.
- Categorical variable encoding
  We applied two preprocessing methods to categorical variables. Label encoding was used to convert fields such as ethnicity and sex into integer values, with missing values handled automatically by the factorise function.

Multi-hot encoding: Multi-hot encoding was utilised for features like comorbidities, which can coincide. The technique allowed for multiple patient conditions to be recorded together while maintaining detailed information.

- Comorbidity clustering and filtering
  We grouped individual comorbidities into clinically relevant clusters (e.g. cardiovascular, gastrointestinal) using Charlson Comorbidity Index categories. We excluded incoherent entries and administrative records, such as procedural notes and irrelevant findings, from the dataset. Multi-hot encoding was applied to the comorbidity categories that resulted from the clustering process.
- Feature aggregation and harmonisation
  The study created composite variables by averaging multiple features with similar clinical interpretations. The BASDAI, BASFI, BASG and ASDAS scores were derived by taking the average of both their original and calculated versions, which minimised data duplication and addressed missing data issues.

To unify the variety in text entries, the ethnicity categories were combined into broader demographic groups (Asian, White, Mixed, Other).

- Handling missing values
  We did not apply mean or median imputation to continuous features. Missingness was managed through variable aggregation where appropriate. Zero-padding filled gaps in the temporal sequences. The missing data of the categorical variables was implicitly encoded during factorisation to maintain signal integrity while enabling tree-based classifiers to work effectively.
- Feature scaling
  Numerical features were standardised to zero mean and unit variance using StandardScaler. This ensured balanced feature contributions during model training.
- Stratified data splitting
  We split the data at the patient level to preserve temporal ordering and prevent data leakage. The dataset was divided into training and test sets at a ratio of 80:20 while maintaining class balance through stratification based on the flare vs. non-flare target variable.
- Feature correlation and redundancy analysis
  Pearson correlation coefficients were calculated between all features and the target variable (Appointment Resource). If necessary, the training process excluded features that showed low or noisy correlations. Triangular correlation heatmaps were utilised to evaluate redundancy among different features.
- Time-step expansion for interpretability
  During the SHAP analysis, we transformed the flattened sequence input back to time-specific features. The SHAP algorithm could evaluate the importance scores of both feature types and their specific temporal positions in sequences, which resulted in detailed model predictions interpretability.

### Modelling strategy

Our modelling approach involved several key phases. The first phase was data preprocessing, which included cleaning and standardising the dataset. This step involved handling missing values, encoding categorical variables using techniques such as factorise, multi-hot encoding, and normalising numerical variables. These steps ensured that the data were in a suitable format for training ML models. We utilised patient blood test results, demographic information, electronic patient-reported outcomes scores, comorbidities, weight and height.

**Figure 1.**
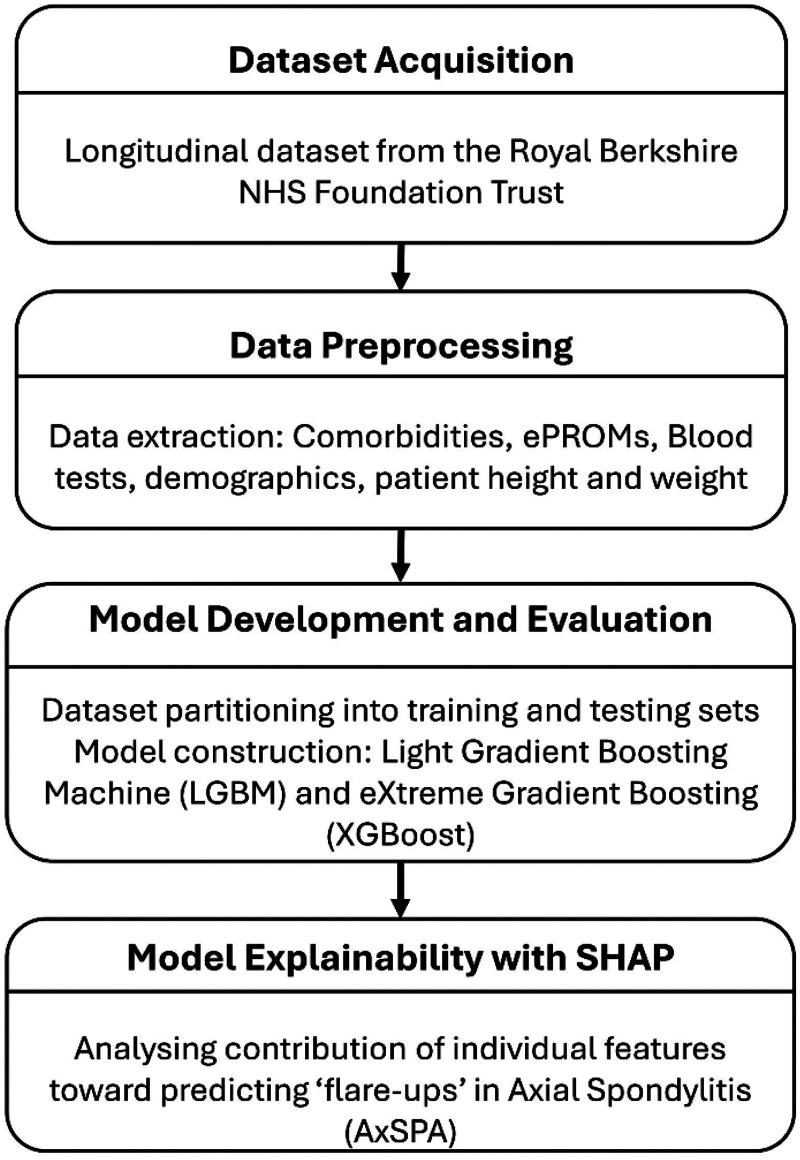
Overview of the machine learning pipeline used to predict future flares in axial spondyloarthritis using longitudinal EHR data. EHR: electronic health record.

In the second phase, we developed LGBM and XGBoost to forecast the risk of flare before their future clinics. The ML model details are provided in supplementary material in section S2. LGBM models were chosen for their ability to capture temporal dependencies in sequential data, which is crucial for understanding disease progression over time. The models were trained on sequences of patient data to capture patterns and trends that may indicate an impending flare. We utilised stratified nested cross-validation techniques to validate the models’ performance and ensure their robustness. Hyperparameters were tuned using randomised search to optimise model performance and its details are provided in supplementary material Table S4. Model performance was evaluated across four-time windows prior to clinic review: 3, 6, 9 and 12 months. The heterogeneous flare-ups prediction pipeline is shown in Figure 1. Details are provided in the supplementary material S1.

### Cross-validation and prevention of overfitting

We implemented a nested cross-validation framework to minimise overfitting and obtain an optimism-corrected estimate of model performance. The dataset was split at the patient level using stratified fivefold outer cross-validation, preserving class balance and preventing temporal leakage. Within each outer training fold, hyperparameters were tuned using RandomisedSearchCV with an internal stratified threefold cross-validation. This two-level structure ensured that tuning occurred only on training data and that each outer fold provided an unbiased evaluation of generalisation. Class imbalance was further addressed using stratified sampling and class-weight adjustments in both LGBM and XGBoost models. For more details, refer to supplementary material S3.2.

Although the dataset included 98 predictors, explicit dimensionality reduction was not required because gradient-boosting models inherently perform feature selection through split-gain optimisation and regularisation. These algorithms down-weight uninformative variables and prioritise features with meaningful predictive signal. SHapley Additive exPlanations (SHAP) analysis confirmed that only a subset of features contributed substantially to model output, indicating that effective dimensionality reduction occurred intrinsically during model training.

### Model performance

A range of metrics was applied to evaluate the performance of the models which ensured that the assessment was thorough. The predictive power of the models was assessed through calculations of accuracy, sensitivity, specificity, precision and the area under the receiver operating characteristic curve (AUROC). The accuracy metric assessed the general correctness level of the model's predictions. Sensitivity (also known as recall) calculated how many actual flares were accurately detected by the model and specificity measured how many non-flares were correctly identified by the model. The positive predictions accuracy made by the model was measured using precision. Geometric-Mean (G-Mean) evaluated model performance across imbalanced datasets by integrating sensitivity with specificity. The detailed information is provided in supplementary material section S3.3.

In addition to discrimination analysis, calibration of both the LGBM and XGBoost models was evaluated using horizon-specific Brier scores and calibration (reliability) plots. For each prediction horizon (3, 6, 9 and 12 months), patients were grouped into deciles of predicted flare risk, and the observed flare frequency within each bin was plotted against the mean predicted probability alongside the ideal 45° line of perfect calibration. Horizon-specific Brier scores were calculated as the mean squared difference between predicted probabilities and observed outcomes, with lower values indicating better calibration.

### SHapley Additive exPlanation analysis

SHAP was derived from game theory and was used after building a ML model. SHAP analysis was conducted post hoc on both global and individual levels. SHAP values quantified how each feature contributed to the model's predictions. This enabled clear interpretation of both global and individual-level feature effects. SHAP explained the actual influence of those features once the model was trained.

### Correlation analysis

We used Pearson correlation coefficient to explore linear relationships between clinical, biochemical, and demographic features and the flare outcome. Details are in supplementary material in section S7.

### Statistical analysis

A chi-square test of independence was calculated on the testing set and training set (the dependent variable represents a value of 1 if the patient experienced a flare and 0 if the patient did not experience a flare) to determine whether categorical patient characteristics were statistically associated with flare occurrence. Details are provided for testing set in Table 1 and for training set is supplementary material in section S9.

**Table 1.** Chi-square associations between categorical demographic variables and flare status.

Categorical feature	Ethnicity label	Chi-squared (X^2^)	Mean	Count	p-value
Ethnicity	Asian	X^2^ = 25.80	0.567	81	1.05 × 10^−^^5^
	Mixed		0.000	2	
	Other		0.266	206	
	White		0.321	946	
	Marital status label		Mean	Count	p-value
Marital status	Divorced	X^2^ = 100.93	0.000	13	6.23 × 10^−^^2^^1^
	Married		0.423	434	
	Separated		0.000	16	
	Single		0.442	319	
	Gender label		Mean	Count	p-value
Gender	Male	X^2^ = 1.04	0.315	787	0.307
	Female		0.345	455	

### Sample size considerations

A formal power analysis was not conducted because the study used all available real-world longitudinal axSpA data, and sample size was determined by disease prevalence and the requirement for complete temporal records. Traditional power calculations are not directly applicable to ML prediction studies; therefore, we ensured robustness through stratified nested cross-validation, class-weight adjustments and randomised hyperparameter optimisation, which together provide reliable performance estimates despite the modest sample size.

### Software and computational environment

All analyses were performed using Python (version 3.11.7) in a Windows environment. ML models were implemented using LGBM (v4.6.0), XGBoost (v3.1.2) and scikit-learn (v1.7.2). Model explainability was conducted using the SHAP package (v0.50.0). Data preprocessing, sequence construction and evaluation metrics were implemented using NumPy (v2.3.5) and pandas (v2.3.3).

## Results

Among 282 patients with axSpA, 175 (63.3%) were males, with a mean (SD) age of 48.2 (14.7) years. Across the 248 patients in the cohort used for model development, there were 5464 clinic visits. Median follow-up time per patient was 3.04 years (interquantile range [IQR] 1.85–4.12), with a median of 16 visits (IQR 8 −13) per patient. The median interval between consecutive visits was 0 days (IQR 0–56 days), reflecting clusters of multiple records on the same calendar day (e.g. clinical assessment and linked tests) alongside longer gaps between clinic appointments. The demographics of the patients are shown in Table 2. During the study period, 100 (35.5%) patients experienced a flare and 182 (64.5%) did not. 175 (62%) patients had radiographic axSpA (r-axSpA). Extra-musculoskeletal manifestations included uveitis (n = 65, 23%), inflammatory bowel disease (n = 17, 6%) and psoriasis (n = 25, 9%).

**Table 2.** Baseline demographic, clinical, and comorbidity characteristics of patients with flare and non-flare status.

	Flare N = 100	No-flare N = 182	Overall N = 282
Age mean/SD	50.46 (15.36)	47.13 (14.29)	48.22 (14.73)
Sex male %	59.64	65.02	63.26
Sex female %	40.35	34.97	36.73
Weight mean/SD (kg)	84.99 (17.94)	88.24 (21.91)	86.95 (20.46)
Height mean (cm)	166.98(17.93)	170.19 (14.54)	168.86 (16.08)
ASDAS mean/SD	1.82 (1.28)	1.78 (1.31)	1.80 (1.29)
BASDAI mean/SD	4.607 (2.446)	3.883 (2.479)	4.106 (2.490)
Ethnicity White %	75.72	77.45	76.89
Ethnicity Asian %	11.54	3.75	6.29
Ethnicity Other %	12.72	18.12	16.36
Ethnicity Mixed %	0.00	0.23	0.16
DRUGS_csDMARD %	6.56	3.39	4.42
DRUGS_bDMARD %	7.25	5.71	6.21
DRUGS_tsDMARD %	0.09	0.14	0.12
DRUGS_Steroids %	0.98	0.47	0.64
Cardiovascular diseases %	32.51	7.29	15.52
Gastrointestinal disorders %	10.36	3.84	5.97
Infections %	18.64	3.63	8.53
Metabolic disorders %	6.95	1.76	3.46
Musculoskeletal disorders %	87.76	52.35	63.91
Neurological disorders %	6.80	0.98	2.88
Respiratory diseases %	27.03	1.96	10.14
Renal and urological disorders %	5.77	0	1.88
Risk factors %	21.55	10.18	13.89

BASDAI: Bath Ankylosing Spondylitis Disease Activity Index; ASDAS: Axial Spondyloarthritis Disease Activity Score.

Prior to their scheduled clinic appointments, we used predictive modelling techniques to anticipate flare and non-flare events based on collected patient data. We divided the dataset into training and testing sets by using data collected at 3 months and 6 months as well as 9 months and 12 months before the clinic visits. The models were evaluated using key performance metrics including accuracy, sensitivity, specificity, precision, AUROC and G-Mean, as summarised in Tables 3 and 4.

**Table 3.** Results of flare prediction in axSpA using LGBM machine learning model.

LGBM	Accuracy	Sensitivity	Specificity	Precision	AUROC	G-mean
3 months within the next clinics	0.846 (95% CI 0.545-0.980)	0.833 (95% CI 0.358-0.995)	0.857 (95% CI 0.421-0.996)	0.833 (95% CI 0.358-0.995)	0.845 (95% CI 0.615-1.000)	0.845 (95% CI 0.647-1.000)
6 months within the next clinics	0.812 (95% CI 0.543-0.959)	0.857 (95% CI 0.421-0.996)	0.777 (95% CI 0.399-0.971)	0.750 (95% CI 0.349-0.968)	0.817 (95% CI 0.593-1.000)	0.816 (95% CI 0.627-1.000)
9 months within the next clinics	0.809 (95% CI 0.580-0.945)	0.875 (95% CI 0.473-0.996)	0.769 (95% CI 0.461-0.949)	0.700 (95% CI 0.347-0.933)	0.822 (95% CI 0.621-1.000)	0.820 (95% CI 0.657-0.983)
12 months within the next clinics	0.772 (95% CI 0.546-0.921)	0.777 (95% CI 0.399-0.971)	0.769 (95% CI 0.461-0.949)	0.700 (95% CI 0.347-0.933)	0.773 (95% CI 0.562-0.984)	0.773 (95% CI 0.596-0.951)

axSpA: axial spondyloarthritis; LGBM: Light Gradient-Boosting Machine; AUROC: area under the receiver operating characteristic curve.

**Table 4.** Results of flare prediction in axSpA using XGBOOST machine learning model.

XGBOOST	ACCURACY	SENSITIVITY	SPECIFICITY	PRECISION	AUROC	G-MEAN
3 months within the next clinics	0.769 (95% CI 0.461-0.949)	0.666 (95% CI 0.222-0.956)	0.857 (95% CI 0.421-0.996)	0.800 (95% CI 0.283-0.994)	0.761 (95% CI 0.487-1.000)	0.755 (95% CI 0.513-0.998)
6 months within the next clinics	0.750 (95% CI 0.476-0.927)	0.714 (95% CI 0.290-0.963)	0.777 (95% CI 0.399-0.971)	0.714 (95% CI 0.290-0.963)	0.746 (95% CI 0.490-1.000)	0.745 (95% CI 0.527-0.963)
9 months within the next clinics	0.714 (95% CI 0.478-0.887)	0.750 (95% CI 0.349-0.968)	0.692 (95% CI 0.385-0.909)	0.600 (95% CI 0.262-0.878)	0.712 (95% CI 0.484-0.958)	0.720 (95% CI 0.526-0.915)
12 months within the next clinics	0.681 (95% CI 0.451-0.861)	0.666 (95% CI 0.299-0.925)	0.692 (95% CI 0.385-0.909)	0.600 (95% CI 0.262-0.878)	0.679 (95% CI 0.443-0.915)	0.679 (95% CI 0.479-0.878)

axSpA: axial spondyloarthritis; XGBOOST: eXtreme Gradient Boosting; AUROC: area under the receiver operating characteristic curve.

LGBM achieved superior discrimination compared to XGBoost. The optimal performance was recorded at 3 months prior to clinic visits showing an accuracy rate of 84.6% (95% CI 0.545–0.980), an AUROC measurement of 0.845 (95% CI 0.615–1.000) and balanced sensitivity 0.833 (95% CI 0.358–0.995) with specificity 0.857 (95% CI 0.421–0.996).

**Figure 2.**
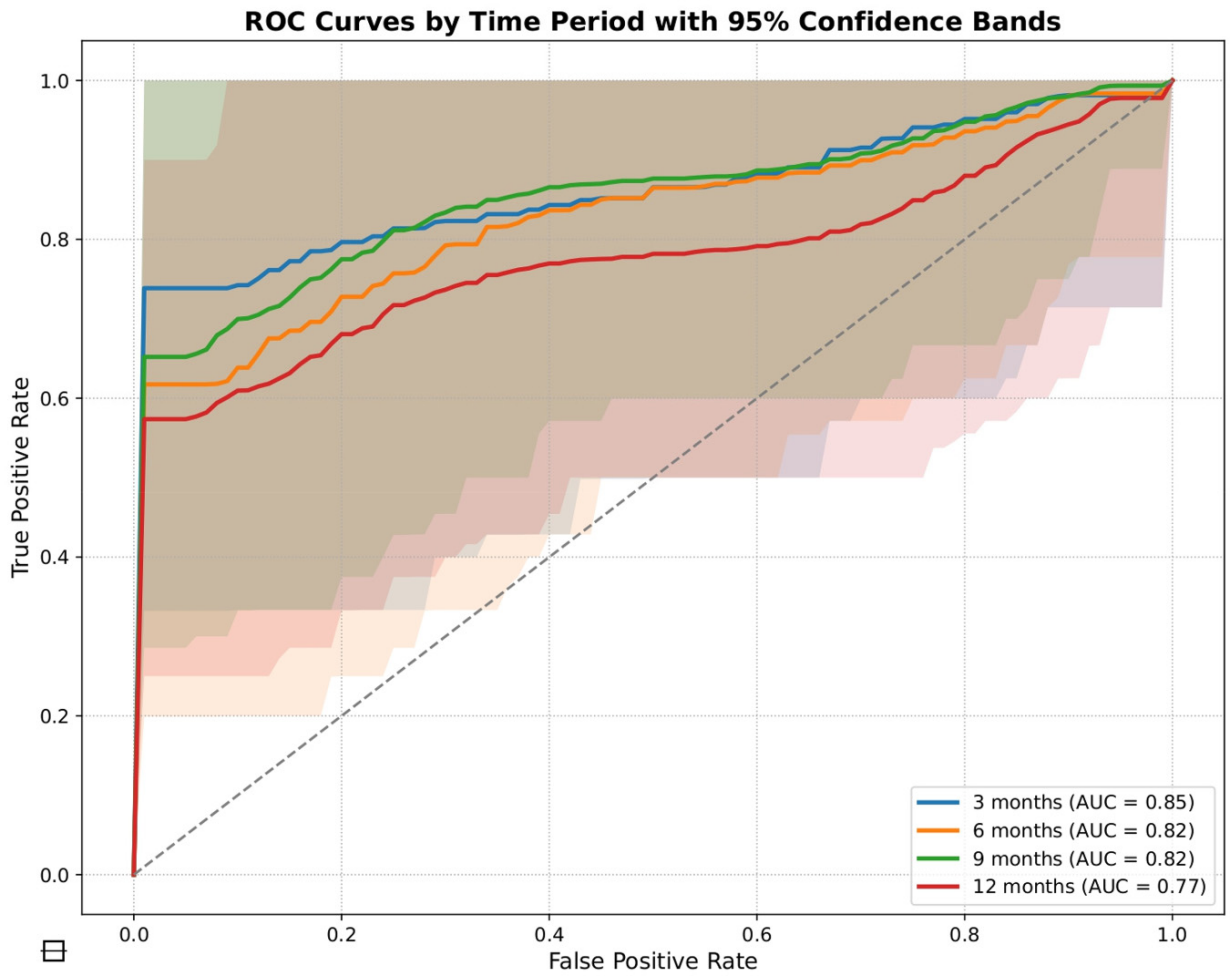
ROC curves showing the discrimination of the LGBM model in predicting flares at 3, 6, 9, and 12 months before clinic visits. LGBM: Light Gradient-Boosting Machine.

Among all prediction horizons, the 3 months window demonstrated the highest AUROC 0.845 (95% CI 0.615–1.000), with the 6- and 9-month window showing slightly lower but still robust discrimination (AUROC: 0.817 and 0.822). The model demonstrated good performance metrics by achieving high precision 0.833 (95% CI 0.358–0.995) and G-Mean 0.845 (95% CI 0.647–1.000), demonstrating its capacity to efficiently separate flare from non-flare cases with minimal trade-offs. Table 3 shows results from the LGBM model which maintained strong performance across every measured time point. The model demonstrated consistent robust performance at 6 and 9 months by maintaining AUROC values above 0.81 and G-Mean values above 0.81 which proves its reliability for medium-term predictions. The AUROC plots for 3, 6, 9 and 12 months with 95% confidence bands until the next clinic visit are shown in Figure 2.

The XGBoost model demonstrated strong performance and is shown in Table 4 especially when predicting at 3 and 6 months before actual clinic appointments. The model demonstrated strong performance at 3 months by reaching an AUROC of 0.761 (95% CI 0.487–1.000) and precision of 0.800 (95% CI 0.283–0.994) while achieving strong specificity of 0.857 (95% CI 0.421–0.996). The AUROC confidence intervals at 12 months are 0.773 (95% CI 0.562–0.984) for LGBM vs 0.679 (95% CI 0.443–0.915) for XGBoost. In the supplementary material, Figure S2 displays the AUROC plots for predicting the next clinic visit at 3, 6, 9 and 12 months.

To facilitate direct comparison between models, Table 5 summarises the performance of LGBM and XGBoost across all prediction horizons. LGBM consistently outperformed XGBoost for every metric and at every time window (3, 6, 9 and 12 months). The largest differences were observed in AUROC, accuracy and G-Mean, particularly at the 12-month horizon where LGBM maintained more robust discrimination. Both models showed expected reductions in performance as the prediction interval increased, but LGBM demonstrated greater stability across all horizons.

**Table 5.** Summary comparison of LGBM and XGBoost performance across prediction horizons.

Metric	Model	3 months	6 months	9 months	12 months
Accuracy	LGBM	0.846	0.812	0.809	0.772
	XGBoost	0.769	0.750	0.714	0.681
Sensitivity	LGBM	0.833	0.857	0.875	0.777
	XGBoost	0.666	0.714	0.750	0.666
Specificity	LGBM	0.857	0.777	0.769	0.769
	XGBoost	0.857	0.777	0.692	0.692
Precision	LGBM	0.833	0.750	0.700	0.700
	XGBoost	0.800	0.714	0.600	0.600
AUROC	LGBM	0.845	0.817	0.822	0.773
	XGBoost	0.761	0.746	0.712	0.679
G-Mean	LGBM	0.845	0.816	0.820	0.773
	XGBoost	0.755	0.745	0.720	0.679

LGBM: Light Gradient-Boosting Machine; XGBoost: eXtreme Gradient Boosting; AUROC: area under the receiver operating characteristic curve.

Calibration performance was generally favourable across the four prediction horizons for both the LGBM and XGBoost models. For the LGBM model, Brier scores were 0.154, 0.165, 0.153 and 0.180 for the 3, 6, 9 and 12 month horizons, respectively, suggesting better calibration at shorter and intermediate intervals, with a modest decline at 12 months.

For the XGBoost, Brier scores were 0.167, 0.169, 0.169, and 0.185 for the same horizons, again indicating acceptable calibration, though with slightly greater miscalibration at the longest horizon.

Calibration curves for LGBM and for XGBoost are shown in supplementary material Figure S3 and S4 that showed close alignment with the ideal 45° line in the low-to-moderate predicted probability range, where most predictions clustered. As expected, both models exhibited greater variability and mild departures from perfect calibration at higher predicted probabilities, reflecting the smaller number of patients contributing to these bins and the greater uncertainty at the extremes. This pattern was visible across all horizons and was most pronounced for the 12-month horizon, where sample sizes were lowest and prediction uncertainty highest.

### Determinants of flare in the predictive model

**Figure 3.**
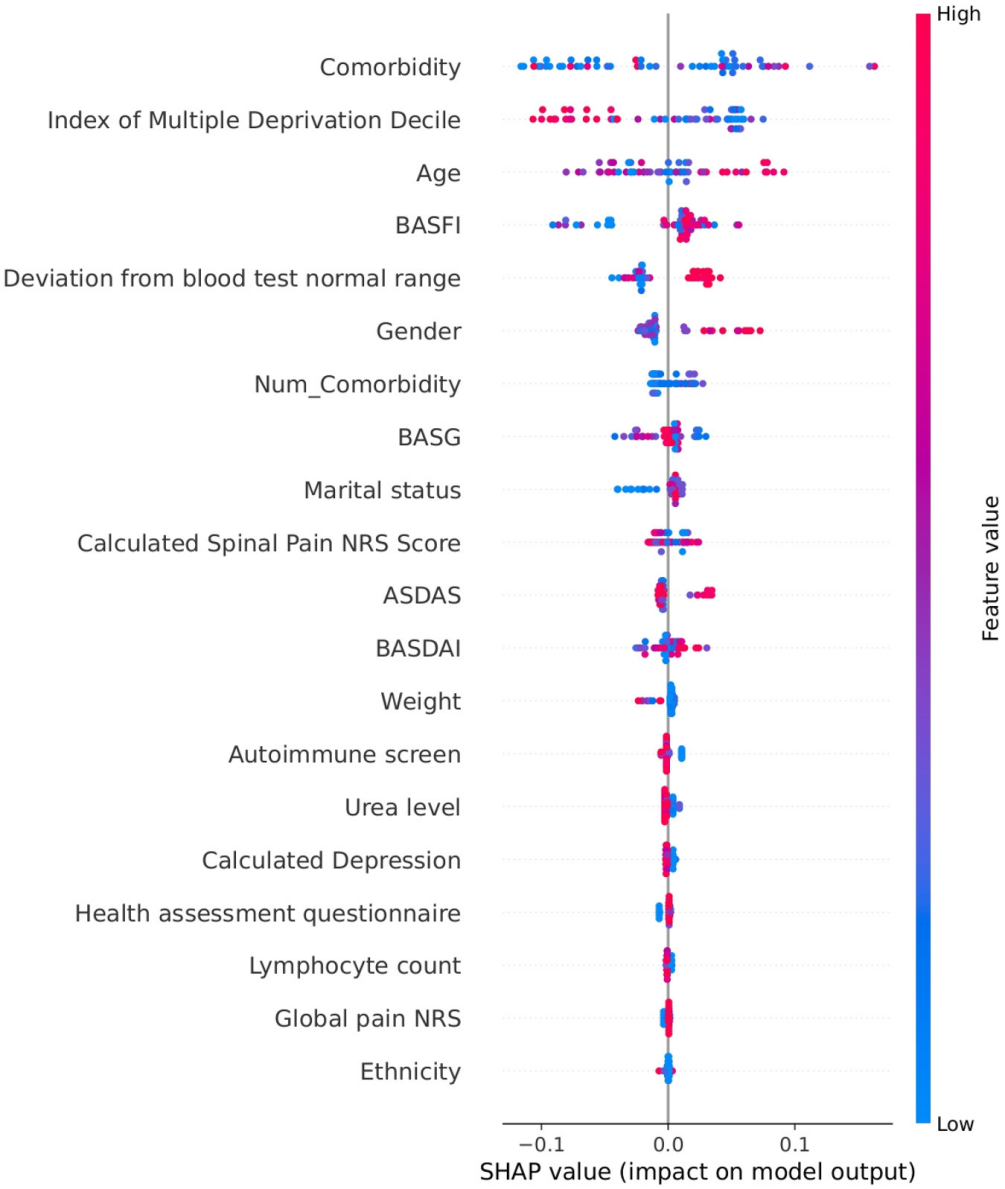
Summary plot showing the most influential features contributing to flare predictions in the LGBM model. LGBM: Light Gradient-Boosting Machine.

The study used SHAP from game theory to determine flare-related factors and explain ML model results. The SHAP summary plot in Figure 3 demonstrates the distribution and significance of the 20 most influential features in an LGBM model's predictions. SHAP values quantify feature impact on predictions and rank features according to their importance. Higher feature values accompanied by positive SHAP values raise the likelihood of flares, whereas negative SHAP values show less probability of flares occurring. The y-axis displays features in order of their impact magnitude, and the colour gradient ranges from blue to red to represent feature values, where blue signifies lower values and red indicates higher values. The model predicted flare likelihood by analysing factors including ethnicity and age as well as marital status alongside functional and disease activity indices including BASDAI, the ASDAS, the Functional Index (BASFI), the Global Assessment (BASG), the Calculated Spinal Pain NRS, socioeconomic status (index of multiple deprivation decile), comorbidity burden and physiological tests results such as lymphocyte count and urea level. Derived features like ‘deviation from blood test normal range’ demonstrate abnormal clinical readings’ role in increasing flare risk. The SHAP summary plot shows each feature's influence on flare risk, indicating variable effects that change according to feature values.

The SHAP summary dot plot reveals multiple critical features in the LGBM model which affect disease flare risk. Reviewing the determinants from highest to lowest ranking, comorbidities had the highest importance (Figure 3). The presence of coexisting comorbidities resulted in increased SHAP values which imply that a greater disease burden heightens flare vulnerability. The number of comorbidities served as a predictor of flares. The index of multiple deprivation decile measured socioeconomic status where higher deprivation levels increased the risk of flares. Beyond disease-specific metrics, age, gender and marital status stood among the highest-ranked features. SHAP values increased with older age which suggested a growing flare risk as people age. Gender and marital status showed moderate influence on flare risk.

The model demonstrated the importance of deviation in blood tests outside the normal range. This includes deviation in haematological, biochemical and autoimmune-related lab values outside the normal range. This created a multifactorial assessment of flare risk. Disease activity measures in axSpA including BASDAI, BASFI, BASG, ASDAS and pain scores were core indicators of flares. These outcomes including the predicted flare risk. The variable SHAP values spanned broad ranges which indicated their strong and consistent impact throughout the patient group. Increased index values led to a higher likelihood of experiencing flare events.

Both mental health conditions and systemic inflammatory responses impacted predictions through markers like calculated depression scores, lymphocyte count and urea level. Ethnicity and body weight showed weaker effects individually but could be important when combined or interacting. The SHAP plot demonstrated that flare risk emerged from multiple factors including disease activity, physiological indicators, demographic context, comorbid burden and social determinants. The model's predictive accuracy benefits from each unique feature which helped identify individuals at high risk of flares.

### Explaining risk of flare of individual patient in the next 3 to12 months

**Figure 4.**
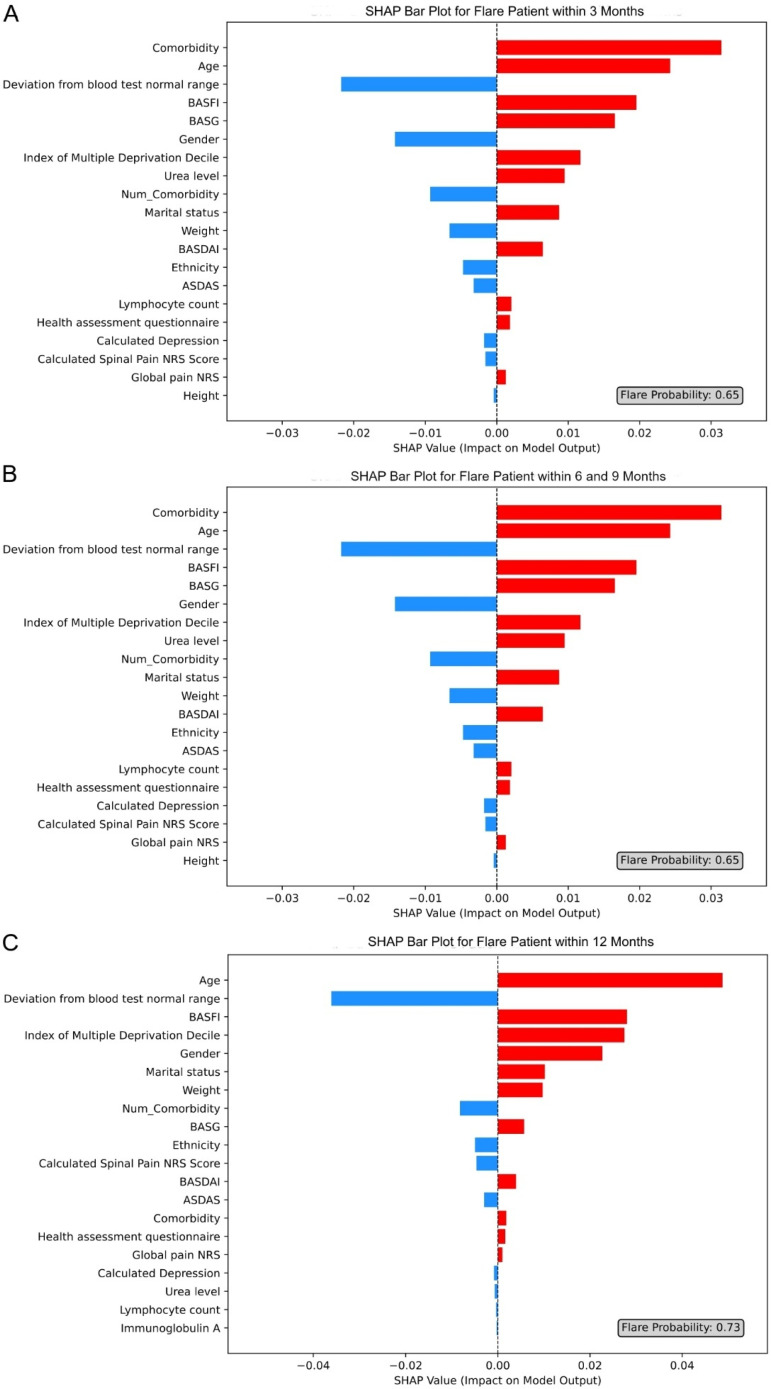
Feature contributions for an individual patient's predicted flare risk across multiple prediction horizons.

Our method was used to explain flare risk predictions in the next 3, 6, 9 and 12 months of individual patients. This was done by identifying essential features that contributed to the predicted flare risk (e.g. blood tests, pain scores, and comorbidities) of different individual patients. The personalised flare risk explanations demonstrate different risk factors of the flare risk across different patients, which helps clinicians understand model decisions of individual patients and create individualised treatment plans. Figure 4 and the supplementary material section S11 provide the information about risk factors that contributed to flare risk prediction of individual patients based on LGBM and XGBoost models.

To further illustrate the model's interpretability at the individual level, Figure 4 shows the SHAP bar plots for an individual flare patient. The SHAP bar plots illustrate the model's interpretability for a patient across different time frames including 3 months and then 6 to 9 months before their clinic visits as well as at 12 months prior. The feature contributions to flare risk remained steady between 3 and 6 to 9 months, with comorbidities, age, blood test deviations and functional scores (BASFI, BASG) each playing equal roles to reach a 0.65 probability prediction. The 12-month plot demonstrated distinct contributing factors with age and immunoglobulin levels playing greater roles and resulting in an elevated flare probability of 0.73. The model demonstrated its ability to track patient risk profile changes through time by identifying different contributing features which allow better understanding of flare risk progression and support proactive clinical decisions.

### Correlation analysis of flare presence on axial spondyloarthritis patients

The analysis of correlations identified multiple features that showed significant relationships with flare status in the test data set and is shown in Table 6 and supplementary material Figure S5. The Pain NRS score demonstrated the strongest positive correlation (r ≈ 0.93, p ≈ 3.22 × 10^−^^4^) which confirms the relationship between patient-reported pain and disease flare occurrences. Albumin demonstrated the highest negative correlation (r ≈ −0.98, p ≈ 2.48 × 10^−^^3^) which indicates that lower albumin levels often linked with systemic inflammation correlated closely with flare episodes. The BASDAI demonstrated a moderate positive correlation (r ≈ 0.36, p ≈ 2.06 × 10^−^^8^) which validated its importance in disease activity measurement. The BASFI demonstrated a lower correlation of approximately 0.16 and a p-value of about 1.8 × 10^−^^2^ which suggests that functional impairment measurements do not always correspond directly with flare risk.

**Table 6.** Correlation analysis on testing set with variable category, variable, coefficient and p-value.

Variable category	Variable	Correlation coefficient	p-value
Patient-reported outcomes and disease activity measures	BASDAI	0.360	2.06 × 10^−^^8^
	BASFI	0.155	1.83 × 10^−^^2^
	BASG	0.335	3.99 × 10^−^^7^
	BASMI	−0.172	5.09 × 10^−^^1^
	Global Likert Pain NRS score	0.927	3.22 × 10^−^^4^
	Spinal pain NRS score	0.234	5.54 × 10^−^^3^
	Calculated swollen joint count	0.395	2.93 × 10^−^^1^
	Calculated tender joint count	0.375	3.19 × 10^−^^1^
Height weight	Height	0.048	1.09 × 10^−^^1^
	Weight	−0.034	2.48 × 10^−^^1^
Blood test	Albumin	−0.983	2.48 × 10^−^^3^
	Deviation from blood test normal range	0.121	6.93 × 10^−^^2^
	Alanine transaminase	−0.167	7.20 × 10^−^^1^
	Alkaline phosphatase	0.695	8.25 × 10^−^^2^
	Antinuclear panel	−0.944	2.12 × 10^−^^1^
	Basophil count	0.084	8.43 × 10^−^^1^
	Bilirubin	−0.216	7.27 × 10^−^^1^
	Calcium adjusted	−0.280	6.48 × 10^−^^1^
	C-reactive protein	0.704	7.70 × 10^−^^2^
	Creatinine	0.285	4.94 × 10^−^^1^
	Eosinophil count	−0.225	5.92 × 10^−^^1^
	Erythrocyte sedimentation rate	0.602	1.14 × 10^−^^1^
	Haematocrit	−0.633	9.16 × 10^−^^2^
	Haemoglobin	−0.705	5.07 × 10^−^^2^
	Immunoglobulin A	0.949	3.73 × 10^−^^3^
	Immunoglobulin G	−0.734	9.62 × 10^−^^2^
	Immunoglobulin M	−0.439	3.83 × 10^−^^1^
	Lymphocyte count	−0.466	2.44 × 10^−^^1^
	Mean cell haemoglobin	−0.263	5.29 × 10^−^^1^
	Mean cell haemoglobin conc	−0.119	7.77 × 10^−^^1^
	Mean cell volume	−0.083	8.45 × 10^−^^1^
	Mean platelet volume	−0.913	8.62 × 10^−^^2^
	Monocyte count	0.286	4.92 × 10^−^^1^
	Neutrophil count	0.593	1.21 × 10^−^^1^
	Phosphate level	−0.950	1.30 × 10^−^^2^
	Platelet count	−0.210	6.16 × 10^−^^1^
	Potassium	0.731	3.93 × 10^−^^2^
	Red blood cell count	−0.725	4.15 × 10^−^^2^
	Red cell distribution width	0.362	4.24 × 10^−^^1^
	Sodium	0.346	4.01 × 10^−^^1^
	Total protein refractometry	−0.981	1.21 × 10^−^^1^
	Urea level	−0.071	8.65 × 10^−^^1^
	White blood cell count	0.591	1.22 × 10^−^^1^
	Index of multiple deprivation decile	−0.004	8.79 × 10^−^^1^
Demographics	Ethnicity	0.056	6.08 × 10^−^^2^
	Age	0.441	2.53 × 10^−^^5^^4^
	Gender	−0.199	1.61 × 10^−^^1^^1^
	Marital status	0.259	1.48 × 10^−^^1^^8^
Comorbidity	Infections	0.029	3.21 × 10^−^^1^
	Cardiovascular diseases	0.106	3.50 × 10^−^^4^
	Gastrointestinal disorders	0.029	3.28 × 10^−^^1^
	Metabolic disorders	0.053	7.5 × 10^−^^2^
	Musculoskeletal disorders	0.090	2 × 10^−^^3^
	Neurological disorders	0.037	2.08 × 10^−^^1^
	Renal and urological disorders	0.037	2.08 × 10^−^^1^
	Respiratory diseases	0.053	7.5 × 10^−^^2^
	Risk factors	0.126	2.30 × 10^−^^5^
Drugs	DRUGS_csDMARD	−0.120	5.53 × 10^−^^5^
	DRUGS_tsDMARD	−0.041	1.68 × 10^−^^1^
	DRUGS_bDMARD	−0.175	3.36 × 10^−^^9^
	DRUGS_Steroids	−0.033	2.61 × 10^−^^1^

BASDAI: Bath Ankylosing Spondylitis Disease Activity Index; NRS: Numerical Rating Score.

The statistical analysis indicated a positive weak but significant correlation between cardiovascular diseases with flare occurrence (r = 0.106, p-value = 3.5 × 10^−^^4^). This showed possible association with conditions including heart failure, myocardial infarction, hypertension, atrial arrhythmias, deep vein thrombosis and chest pain. Musculoskeletal disorders demonstrated a weak but significant correlation (r = 0.091, p-value = 2.38 × 10^−^^3^). This may be related to conditions including osteoarthritis, degenerative joint disorders and fibromyalgia. The weak correlation between metabolic disorders (r = 0.053, p-value = 7.52 × 10^−^^2^) such as diabetes, obesity, hypothyroidism and respiratory diseases (r = 0.053, p-value = 7.51 × 10^−^^2^) such as asthma, COPD indicate possible contribution to flare risk. These results demonstrate the complex interaction between physiological disease burden and systemic comorbidities in predicting flare risk. The association between Immunoglobulin A and phosphate level with flare status yielded statistically significant p-values but additional research is needed to determine its clinical relevance. The details of correlation information are provided in supplementary material in section S7.

Three categorical predictors (ethnicity, marital status, and gender) were included in the analysis (refer Table 1) and for training set analysis refer supplementary material section S9. Ethnicity was a statistically significant predictor (X^2^ = 25.80, p-value = 1.05 × 10^−^^5^). The subgroups with the largest proportion of flares were those of Asian race (Mean = 0.567, Count = 81), followed by White (Mean = 0.321, Count = 946) and Other (Mean = 0.266, Count = 206). The Mixed group did not experience any flares; however, the sample size was very small (Count = 2).

Marital status was also statistically significantly associated with flare outcome (X^2^ = 100.93, p-value = 6.23 × 10^−^^2^^1^). The groups with the largest proportion of flares were those that were single (Mean = 0.442, Count = 319) followed closely by married groups (Mean = 0.423, Count = 434). Both the divorced group (Mean = 0.000, Count = 13) and Separated group (Mean = 0.000, Count = 16) did not experience any flares; however, the sample sizes of these groups were small.

Gender was not a statistically significant predictor (X^2^ = 1.04, p-value = 0.307). The Female group had a higher mean flare rate (Mean = 0.345, Count = 455) compared to the male group (Mean = 0.315, Count = 787). However, this result was not statistically significant. These results indicate that, for the test cohort, ethnicity and marital status were associated with flare occurrence but gender did not have a statistically significant effect on flare risk.

Medication use data were included among the 98 input variables used to train the ML models. In the correlation analysis (Supplementary Section S7.1), treatment with biologic DMARDs (bDMARDs) showed a modest negative correlation with flare occurrence (r = –0.124, *p* < 0.001), as did conventional synthetic DMARDs (csDMARDs) (r = –0.094, *p* < 0.001). The use of steroids had a weaker and non-significant correlation (r = –0.048, *p* = 0.11). In the SHAP feature importance ranking, drug-related variables did not appear among the top 20 predictors of flare. This suggests that, while therapy may contribute to flare risk mitigation, it did not have a dominant effect on short-term flare prediction relative to disease activity, comorbid burden or biochemical markers.

### Feature to feature correlation analysis

**Figure 5.**
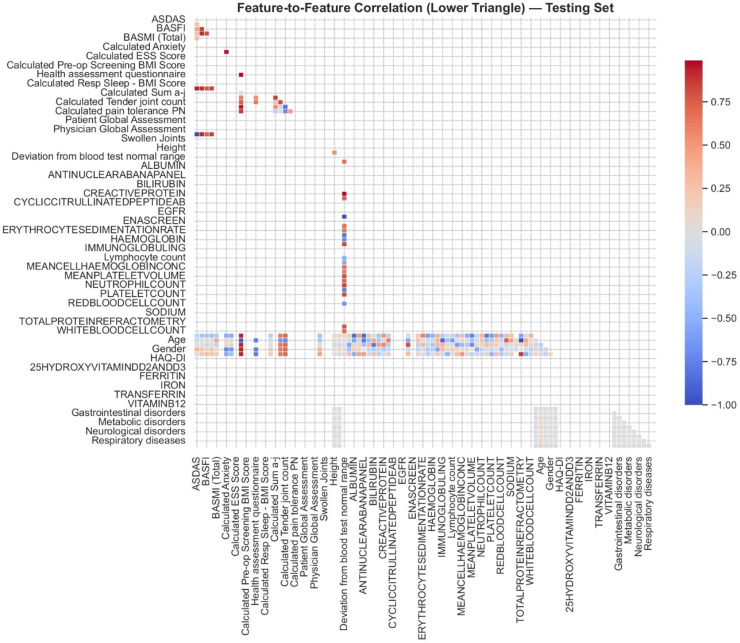
Correlation matrix illustrating relationships among clinical, laboratory, and demographic variables in the test set.

The lower triangular Pearson correlation matrix for the testing set demonstrated the feature interrelationships relevant to axSpA and this is shown in Figure 5. The heat map employed a diverging colour scale to indicate the strength and direction of correlations: A strong positive correlation (r ≈ + 1) is represented by dark red while dark blue indicates a strong negative correlation (r ≈ −1) and negligible correlations (r ≈ 0) appear white. The analysis revealed multiple groups of features that showed high correlation with each other. Inflammatory markers showed strong interdependence. The link between CRP levels and blood test deviations from normal range showed an extremely high correlation value (r ≈ 0.999) which confirmed CRP's key function in systemic inflammation during flare-ups. A strong inverse correlation existed between age and both mean platelet volume and total protein refractometry (r ≈ −0.995 and −0.982) which demonstrates recognised age-related changes in blood and protein metabolic functions. The details of feature-to-feature correlation analysis are provided in supplementary material in section S8.

## Discussion

We conducted predictive modelling to forecast flare and non-flare events in the next 3 to 12 months. Our predictive model demonstrated 84.6% to 77.2% accuracy predicting flare and non-flare events in the next 3 to 12 months. Previous studies have used ML models to predict radiographic progression in axSpA rather than flare-ups by analysing clinical and radiographic data which could be indirectly related to disease activity.^8,9^ The novelty of this study was that it used the combination of EHR-based modelling, multiple time windows and explainable outputs to predict flare risk.

Multiple key features that affected the model's flare risk predictions were identified. The most influential features in predicting flare risk included comorbidity burden, age, functional and disease activity indices (BASDAI, BASFI, BASG, ASDAS), and laboratory deviations from normal ranges. From a clinician's perspective, the fact that comorbidity burden emerged as the strongest predictor aligns with everyday experience in managing complex axSpA patients. Comorbidities, particularly cardiovascular and musculoskeletal conditions, are likely to reflect systemic inflammation, impaired functional reserve and broader disease vulnerability. Cardiovascular diseases (including heart failure, myocardial infarction, hypertension, atrial arrhythmias, deep vein thrombosis and chest pain), musculoskeletal conditions (including spine disorders, back pain, fibromyalgia, injuries) and risk of vulnerability (including pressure sore risk, falls and frailty) demonstrated significant correlation with the flare risk. The cumulative effect of comorbidities, especially when combined with other risk factors, results in a higher overall disease burden and a greater likelihood of disease flares.^10,11^

The risk of experiencing a flare correlated with both marital status and socioeconomic position which was determined using the index of multiple deprivation decile. Single or separated individuals had higher SHAP values than married individuals. This may reflect the protective role of social support in managing disease, promoting treatment adherence and reducing stress-induced flare triggers, while the associations may also reflect unmeasured behavioural or socioeconomic confounders including social isolation. Patients in deprived regions could face increased flare risk because of reduced healthcare access and elevated stress levels.^12,13^ Gender and ethnicity demonstrated smaller effects, yet these differences could indicate variations in healthcare treatment and disease manifestation.

Disease activity measures including BASDAI, ASDAS, BASFI and BASG together with patients’ age showed strong effect on flare risk predictions. The analysis demonstrated that higher scores on these indices combined with older age correlated with increased flare risk. This aligns with previous research demonstrating elevated inflammation, functional limitation and cumulative disease burden through time.^14,15^ Patient-reported pain scores including the calculated spinal pain NRS and global pain NRS demonstrated significant influence. This confirms that patient-reported outcomes are important in flare prediction.^16,17^ The analysis revealed that mental health conditions especially depression played a significant role in increasing flare risk. This demonstrated the important connection between psychological health and disease experience.^18,19^

The lab values including lymphocyte count along with urea showed moderate predictive power. This demonstrated their possible involvement in inflammation and immune responses and requires further study. Deviation from blood test normal ranges (calculated as a proportional distance outside of the reference range and applied consistently across all blood parameters) also contributed to flare prediction significantly. The strong correlation between CRP levels and abnormal blood test findings confirms CRP's established utility as a sensitive systemic inflammation biomarker. The elevated CRP levels found in inflammatory conditions during disease flare-ups is supported by previous studies.^20^ The strong correlation indicates that a change in the CRP could serve as both a standard inflammation marker and a predictive tool for flares^21^ . The negative correlation with albumin levels fits with the model of raised CRP to albumin ratio (CAR) in active axSpA. Studies have shown the rise in CAR was associated with increased disease activity in axSpA.^22,23^ The laboratory results should be interpreted with adjustments for age to ensure accurate clinical practice outcomes. Although medications including bDMARDs, csDMARDs and steroids were included in model training, their relative contribution to flare prediction was limited compared to other features. This may reflect several factors. First, medication use often correlates with disease severity and may be escalated during or following a flare, making temporal disentanglement difficult in cross-sectional feature sets. Second, in our dataset, drug exposure was recorded as a binary feature (i.e. currently prescribed or not), which does not capture dose, treatment duration, recent changes or adherence. These limitations may attenuate the observed impact of medications in SHAP analysis and correlation measures. The negative correlations observed for bDMARDs and csDMARDs with flare occurrence support their protective effect, in line with clinical evidence. The lack of stronger predictive power from drug variables in the current model suggests that future iterations should explore time-varying exposure and causal inference frameworks to better understand drug–flare relationships. Inclusion of detailed treatment timelines and response trajectories could enhance flare risk stratification and personalised treatment recommendations.

The correlation study demonstrated that flare status in axSpA involved multiple dimensions which include clinical assessments together with biochemical markers as essential indicators. Data shows that flares in axSpA patients are closely linked to systemic inflammation and symptom burden through correlation with patient-reported pain and disease activity indices. The correlation analysis supported these findings, demonstrating meaningful associations between pain, hypoalbuminemia, comorbidities and flare risk even when correlation coefficients were modest. Our correlation analysis revealed that several variables had statistically significant but low Pearson correlation coefficients with flare status. This can arise in large datasets or datasets with consistent but subtle effects. For instance, in our study, comorbidities such as cardiovascular or musculoskeletal disorders may individually have a small impact on flare risk, but collectively, their consistent influence across the dataset leads to statistically significant results. Recognising these small but significant signals is essential for multifactorial prediction models like ours. This observation reinforces the need to integrate multiple features within ML models, where additive and interactive effects between weakly correlated features can produce high predictive accuracy. It also justifies our use of SHAP to explore complex feature contributions beyond linear correlations.

Recent methodological guidance for AI-based prediction models emphasises transparent reporting and structured evaluation. The TRIPOD-AI/TRIPOD + AI statement outlines updated standards for reporting ML prediction models,^24^ including clear specification of predictors, handling of missing data, prevention of data leakage and the use of explainability tools. Our modelling approach-patient-level data splitting nested cross-validation, and SHAP-based interpretation-align closely with these recommendations, although formal TRIPOD-AI mapping will be incorporated in future external validation studies. Likewise, PROBAST-AI, an extension of the PROBAST tool for assessing risk of bias in AI prediction models, highlights domains such as participant selection, predictor definition and overfitting control. Our design adheres to these principles through rigorous temporal separation, stratification and hyperparameter optimisation, but future multicentre validation will allow structured PROBAST-AI assessment to further establish model robustness.^25^

The growing use of explainable machine learning in healthcare has highlighted SHAP as one of the most reliable tools for interpreting complex models in chronic disease prediction. Recent reviews show increasing adoption of SHAP to characterise patient-specific risk drivers and support clinical decision-making across specialties, including cardiometabolic and inflammatory diseases.^26,27^ Within rheumatology, emerging explainable models such as deep-learning frameworks for predicting rheumatoid arthritis disease activity or treatment response demonstrate the value of combining predictive accuracy with interpretable output.^28^ Comparable applications in axSpA remain limited, with most prior studies focusing on diagnosis or radiographic progression rather than flare forecasting.^29^ Our work extends this field by providing a real-world, EHR-based, explainable flare-risk model capable of highlighting the specific clinical, biochemical and comorbidity features contributing to individual patient trajectories, supporting future treat-to-target digital pathways.

The strength of our study is that this was taken from real-world clinic data. All patients were followed up through the study period. They had also confirmed flares or non-flares by a Consultant Rheumatologist, improving validation of the cohort studied. We acknowledge that incorporating clinician judgement introduces an element of subjectivity; however, this mirrors real-world flare recognition and increases the model's external relevance.

This study integrated clinical data metrics with physiological indicators and psychosocial factors alongside laboratory values to develop an extensive model for predicting flare risks. The model was able to identify personalised predictors by analysing deviations from normal blood test results and demographic factors including gender and ethnicity. A sophisticated methodology that merged standard indices with extensive contextual information produced more accurate flare predictions. The study outcomes validated a multidimensional disease management approach that supported early detection of high-risk patients and enabled tailored interventions to reduce both the severity and frequency of flare-ups.

The limitations of our study are the small sample size. We also did not have radiographic or imaging data in our study. This was a single-centre study and external validation will be required to validate the model before it is used in clinical pathways. This modest sample size reflects both the rarity of axSpA (∼1% prevalence) and the strict data completeness criteria required for longitudinal ML analysis. While a larger dataset may offer more statistical power and generalisability, our focused and well-curated cohort ensured high data quality, precise flare adjudication and real-world applicability. We implemented multiple strategies to address possible biases which limited data might create. Our initial strategy incorporated stratified nested K-Fold cross-validation which preserved class distribution across all folds to improve performance estimate accuracy. Our LGBM and XGBoost model incorporated class-weighting to prioritise underrepresented classes which helps tackled the common class imbalance problem found in clinical datasets. The study used randomised search cross-validation to tune hyperparameters across multiple folds which minimised the likelihood of overfitting to a single training subset. We improved robustness by aggregating performance metrics from all cross-validation folds which gave us a more comprehensive evaluation of model accuracy. The model's predictive performance was assessed over several clinically important time periods (such as 3, 6, 9 and 12 months before clinic visits) to avoid temporal bias in any specific time frame. We used SHAP-based explainability exclusively on correctly predicted samples to prevent false interpretations caused by noise or misclassification. Our methodological safeguards worked together to minimise bias while boosting both the interpretability and reliability of our research conclusions even with limited sample sizes. Further work includes using advanced data processing methods to handle missing values to improve accuracy, especially for predictions of 12 months and further ahead.

**Figure 6.**
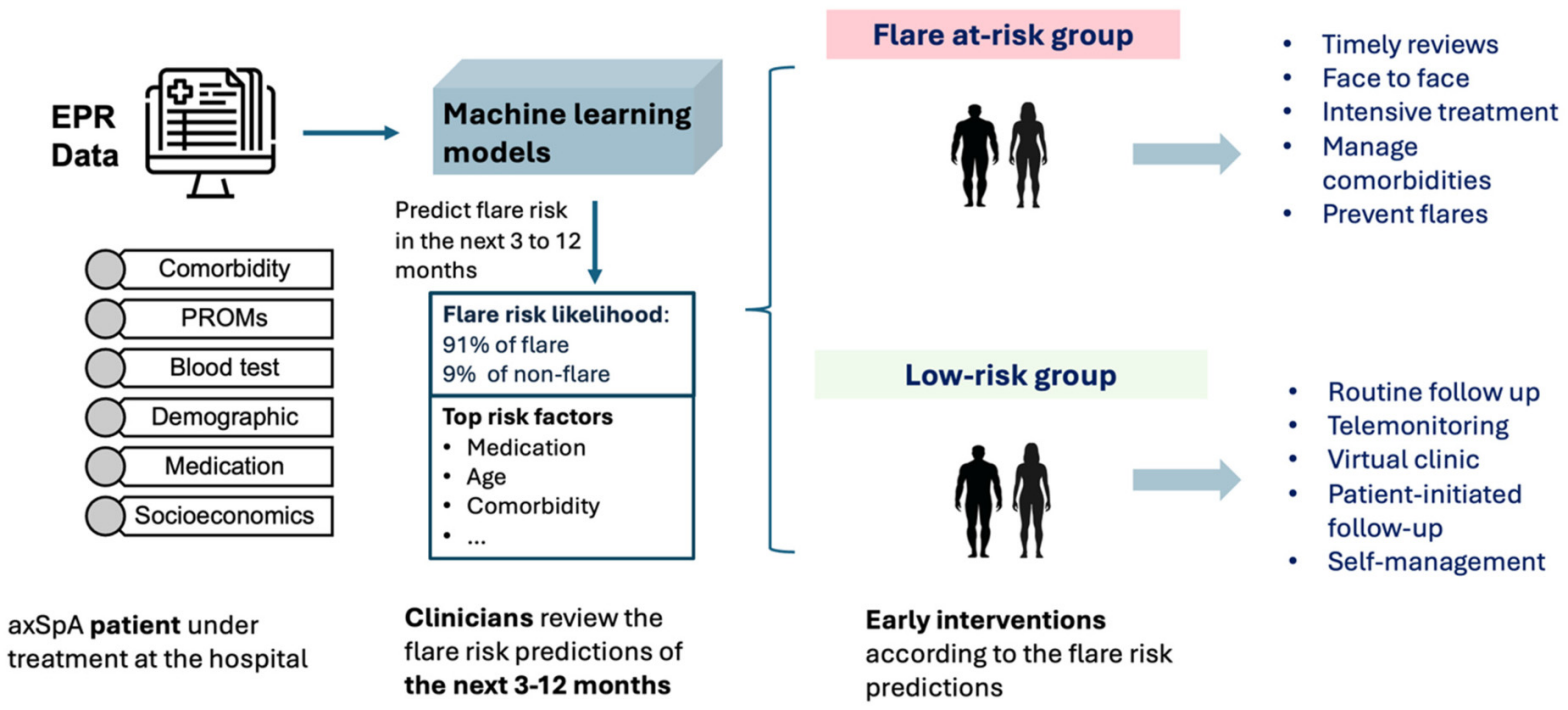
Conceptual illustration of how flare-risk predictions can support proactive clinical management in axSpA. axSpA: axial spondyloarthritis.

Our model identified that flare risk prediction may help clinicians in recognising if patients are at risk of a flare-up. With the informed risk prediction presented early and more accurately, patients could be booked into an early appointment with a specialist so that early interventions can be put in place to prevent flare-ups in a timely way (Figure 6). Clinicians can identify patients at high risk of flare-up so interventions such as intensifying or changing medication can be in place before flare happens. This can lead to better outcomes for axSpA patients in the long term.

## Conclusion

In conclusion, our study showed that it is possible and clinically useful to use ML with routinely collected data to anticipate flares in axSpA. The model integrates biologic, functional, psychosocial and social determinants to deliver personalised predictions that can enhance clinical decision-making and patient outcomes.

## Supplemental Material

sj-docx-1-dhj-10.1177_20552076261433513 - Supplemental material for Explainable machine learning for predicting disease flares in axial spondyloarthritis: A real-world electronic health record-based pilot study Supplemental material, sj-docx-1-dhj-10.1177_20552076261433513 for Explainable machine learning for predicting disease flares in axial spondyloarthritis: A real-world electronic health record-based pilot study by Antoni Chan, Pradip Moon, Weizi Li and Eghosa Bazuaye in DIGITAL HEALTH
