# Supplementary material for: Explainable machine learning for predicting disease flares in axial spondyloarthritis: A real-world electronic health record-based pilot study
Source: Digit Health. 2026 Apr 25;12:20552076261433513. doi: 10.1177/20552076261433513 (PMC13129351; doi:10.1177/20552076261433513)
Supplement: sj-docx-1-dhj-10.1177_20552076261433513 - Supplemental material for Explainable machine learning for predicting disease flares in axial spondyloarthritis: A real-world electronic health record-based pilot study [file sj-docx-1-dhj-10.1177_20552076261433513.docx]

Supplementary Material

[S1 Heterogenous flare-ups prediction pipeline 1](#_Toc1997874508)

[S2.1 Light Gradient Boosting Machine (LGBM) 2](#_Toc70871344)

[S2.2 eXtreme Gradient Boosting (XGBoost) 2](#_Toc1852802677)

[S3 Model selection and evaluation 3](#_Toc1103046405)

[S3.1 Random hyperparameter search 3](#_Toc798587690)

[S3.2 Nested and Stratified cross-validation 3](#_Toc1379413511)

[S3.3 Performance metrics 4](#_Toc287086258)

[S4 Independant variables 5](#_Toc1363356951)

[S5 AUROC Plot 7](#_Toc1688639195)

[S7 Correlation Plot 10](#_Toc847470407)

[S7.1 Correlation analysis on training set 12](#_Toc484930082)

[S8 Feature-to-feature correlation plot 17](#_Toc389414893)

[S9 Chi-square analysis on training set 20](#_Toc2021763031)

[S10 SHAP Plot of variable importance for flare prediction 22](#_Toc205669131)

[S11 Individual patient SHAP bar plot 26](#_Toc1810607173)

[S12 Supplementary dataset 33](#_Toc1651000649)

[S13 Hyperparameter optimisation 34](#_Toc17981229)

[S14 References 35](#_Toc1020559719)

## S1 Heterogenous flare-ups prediction pipeline

This study obtained its longitudinal dataset from the Royal Berkshire NHS Foundation Trust. From the Trust’s SQL Server database, we extracted comorbidity information along with electronic patient-reported outcome measures (ePROMs), blood test results and demographic variables, as well as patient height and weight. After acquiring the raw data, we performed preprocessing to achieve a standardised format according to the structured data section guidelines. The dataset was optimised for machine learning applications by standardising patient-reported outcomes such as BASDAI, BASFI, BASG, and ASDAS, together with blood test results and demographic information, while grouping comorbidities into clinically relevant categories.

The dataset was then partitioned into training and testing sets using an 80% to 20% ratio while ensuring class balance through stratified sampling. The model development process employed Light Gradient Boosting Machine (LGBM) and Extreme Gradient Boosting (XGBoost) classifiers with hyperparameter optimisation conducted through randomised search in a 5-fold nested stratified cross-validation setup to achieve the highest possible area under the receiver operating characteristic curve (AUROC). After training the models with the complete training dataset, we assessed their performance on an independent test set to evaluate their prediction accuracy for flare-ups in axial spondyloarthritis patients.

SHAP (SHapley Additive exPlanations) was used after model training to analyse the contribution of individual features toward predicting flare-ups. The SHAP bar plots demonstrated each feature's average absolute impact, and the dot plots displayed the direction and distribution of effects throughout the patient population. Our explainability framework helped us recognise essential variables influencing model performance, which deepened our understanding of disease activity drivers to better personalise disease management and care planning, as shown in Figure 1.

## S2 Machine learning methods

We used Light Gradient Boosting Machine (LGBM) and eXtreme Gradient Boosting (XGBoost) algorithms to forecast flare-ups for ankylosing spondylitis patients. The sections that follow contain detailed information about the modelling approach.

### S2.1 Light Gradient Boosting Machine (LGBM)

For predicting flare-ups in axial spondyloarthritis patients we used the LGBM algorithm which represents an advanced form of Gradient Boosted Decision Trees (GBDT). LGBM builds decision trees in a sequential manner where every new tree aims to correct the prediction errors left by the prior trees. The sequential learning approach enables the model to target predictive errors that exhibit the highest values to enhance its general performance.

LGBM uses a histogram-based method to determine split points for continuous variables which results in faster training speeds and reduced memory usage. The tree construction process chooses features that maximise information gain to create optimal splits which produces more precise models [1].

This study used randomised search with stratified 5-fold cross-validation to optimise hyperparameters including maximum depth (max_depth), number of leaves (num_leaves), minimum child weight (min_child_weight), minimum child samples (min_child_samples), number of estimators (n_estimators), learning rate (learning_rate), feature fraction (colsample_bytree), subsample fraction (subsample), L1 and L2 regularisation terms (reg_alpha, reg_lambda), and class weight adjustment (scale_pos_weight) and its values shown in Table S1. SHapley Additive exPlanations (SHAP) increased model interpretability by revealing how temporal clinical features, and comorbidity clusters contribute to predicting flare events.

### S2.2 eXtreme Gradient Boosting (XGBoost)

XGBoost represents an enhanced version of the gradient boosting decision tree (GBDT) algorithm, which targets improved efficiency and scalability along with high performance. The algorithm sequentially trains multiple decision trees, with each subsequent tree designed to address the mistakes of its predecessors. XGBoost achieves better computational performance and efficient processing of large datasets using parallelisation and memory optimisation techniques, unlike traditional boosting algorithms [2].

The XGBoost model underwent training with Stratified K-Fold cross-validation while Randomised Search determined the optimal hyperparameters. The study achieved precise model adjustments through tuning max_depth, learning_rate, n_estimators, colsample_bytree, subsample, gamma, reg_alpha, reg_lambda, and min_child_weight which controlled model complexity and learning rate as well as feature sampling and regularisation strength and its parameter values shown in Table S1. The model underwent specific optimisation to achieve binary classification through the binary logistic objective. Class imbalance was managed by setting the scale_pos_weight parameter to match the ratio of non-flare events to flare events within the training dataset. The contribution of clinical and demographic variables in predicting flare events was evaluated through SHAP (SHapley Additive exPlanations) analysis for feature importance interpretation. SHAP-based methods enabled systematic evaluation of model explainability and feature contributions without employing conventional recursive feature elimination procedures.

## S3 Model selection and evaluation

To find the best hyperparameters for the model, we used random search hyperparameter optimisation coupled with stratified cross-validation, as described below.

### S3.1 Random hyperparameter search

The optimal parameter combinations for the model used in the outer training set were determined through random search hyperparameter optimisation. We evaluated the model's performance by comparing its best estimator and score against the best hyperparameter. The random search algorithm utilises probability distributions to assign values to each parameter autonomously. The search space represents a bounded domain where each point shows different hyperparameter values, and the domain undergoes arbitrary sampling. Supplementary Table S1 displays the optimal hyperparameters.

### S3.2 Nested and Stratified cross-validation

Model development followed a nested cross-validation framework comprising an outer stratified 5-fold cross-validation for performance estimation and an inner stratified 3-fold cross-validation for hyperparameter tuning. In the outer loop, patients were partitioned into five folds with preserved flare status distribution. Each fold served once as an external test set, while the remaining folds formed the training set. Within each outer training set, RandomisedSearchCV was used for hyperparameter optimisation using an internal 3-fold stratified CV. The best model from the inner loop was then evaluated on the corresponding unseen outer fold.

This hierarchical CV design prevents information leakage between tunning and evaluation, providing an optimism-corrected estimate of generalisation performance and reducing the risk of overfitting inherent to high-dimensional clinical datasets.

### S3.3 Performance metrics

To check the performance of LGBM and XGBoost machine learning models we employed following performance metrics.

- Accuracy

This metric calculates the ratio of correctly predicted outcomes to the total number of cases [4].

*Accuracy = (TP + TN) / (TP + TN + FP + FN)*

where, TP is true positive, TN is true negative), FP is false positive) and FN is false negative.

- Sensitivity (Recall or True Positive Rate)

This metric calculates the ability of the model to detect positive instances correctly. High sensitivity means few positive cases are missed [4].

Sensitivity = TP / (TP + FN)

where, TP is true positive and FN is false negative.

- Specificity (True Negative Rate)

This metric calculates the model’s ability to correctly identify negative instances. High specificity means few negative cases are wrongly classified as positive [4].

*Specificity = TN / (TN + FP)*

where, TN is true negative, and FP is false negative.

- Precision

The metric evaluates the ratio between true positive cases and all predicted positive cases. Focuses on the correctness of positive predictions [4].

*Precision = TP / (TP + FP)*

where, TP is true positive, and FP is false positive.

- AUC

The AUROC value shows how often the classifier correctly ranks random positive cases above random negative cases. Higher AUROC indicates better model separation.

$$AUC = \int_{0}^{1} TPR\left( t \right) FPR\left( t \right) dt$$

where TPR is true positive rate and FPR is false positive rate and t is a varying parameter in [0,1] [4].

- G-Mean

The G-Mean metric assesses classification performance by evaluating the balance between sensitivity and specificity with a higher G-Mean showing effective classification for both classes [4].

$G-Mean=\sqrt{Sensitivity\times Specificity}$

## S4 Independant variables

The table shows the independent variables are used in LGBM and XGBoost machine learning modelling.

| **Independent Variables** |
| --- |
| MY_UNIQUE_IDENTIFIER |
| ASDAS |
| BASDAI |
| BASFI |
| BASG |
| BASMI (Total) |
| Calculated ASDAS CPR Partial |
| Calculated ASDAS ESR Partial |
| Calculated Alcohol consumption total units |
| Calculated Anxiety |
| Calculated BAS-G Score |
| Calculated BASDAI Score |
| Calculated BASFI Score |
| Calculated Depression |
| Calculated ESS Score |
| Calculated FRAX |
| Calculated HAQ-DI |
| Calculated Likert Pain NRS Score |
| Calculated Pre-op Screening BMI Score |
| Calculated Pre-op Screening Yes/No Score |
| Calculated PsAID-12 Score |
| Calculated QRISK3 |
| Calculated RAPID-3 |
| Calculated Resp Sleep - BMI Score |
| Calculated Spinal Pain NRS Score |
| Calculated Sum a-j |
| Calculated Swollen joint count |
| Calculated Tender joint count |
| Calculated global estimate PTGE |
| Calculated pain tolerance PN |
| FACIT-F |
| Patient Global Assessment |
| Patient Global Pain VAS |
| Physician Global Assessment |
| Spinal Pain VAS |
| Swollen Joints |
| Tender Joints |
| HEIGHTLENGTHMEASURED |
| WEIGHTMEASURED |
| Deviation from blood test normal range |
| ALANINETRANSAMINASE |
| ALBUMIN |
| ALKALINEPHOSPHATASE |
| ANTINUCLEARABANAPANEL |
| BASOPHILCOUNT |
| BILIRUBIN |
| CALCIUMADJUSTED |
| CREACTIVEPROTEIN |
| CREATININE |
| CYCLICCITRULLINATEDPEPTIDEAB |
| DSDNAELISA |
| EGFR |
| EGFRCKDEPI |
| ENASCREEN |
| EOSINOPHILCOUNT |
| ERYTHROCYTESEDIMENTATIONRATE |
| HAEMATOCRIT |
| HAEMOGLOBIN |
| IMMUNOGLOBULINA |
| IMMUNOGLOBULING |
| IMMUNOGLOBULINM |
| LYMPHOCYTECOUNT |
| MEANCELLHAEMOGLOBIN |
| MEANCELLHAEMOGLOBINCONC |
| MEANCELLVOLUME |
| MEANPLATELETVOLUME |
| MONOCYTECOUNT |
| NEUTROPHILCOUNT |
| PHOSPHATELEVEL |
| PLATELETCOUNT |
| POTASSIUM |
| REDBLOODCELLCOUNT |
| REDCELLDISTRIBUTIONWIDTH |
| SODIUM |
| THYROIDSTIMULATINGHORMONE |
| TOTALPROTEINREFRACTOMETRY |
| UREALEVEL |
| WHITEBLOODCELLCOUNT |
| COMORBIDITY |
| Drugsbetweendates |
| Index of Multiple Deprivation Decile |
| AGE_AT_APPOINTMENT_DATE |
| ETHNICITY |
| PERSON_GENDER_CODE_DESC_CDS |
| PERSON_GENDER_CODE_LOCAL |
| PERSON_MARITAL_STATUS_CODE_LOCAL |
| DIAGNOSES |
| HAQ-DI |
| PsAID12 |
| 25HYDROXYVITAMINDD2ANDD3 |
| ALPHAFETOPROTEIN |
| FERRITIN |
| FOLATE |
| IRON |
| RHEUMATOIDFACTOR |
| TRANSFERRIN |
| TRANSFERRINSATURATION |
| VITAMINB12 |

**Table S1.** Independant variables used in machine learning modelling

## S5 AUROC Plot


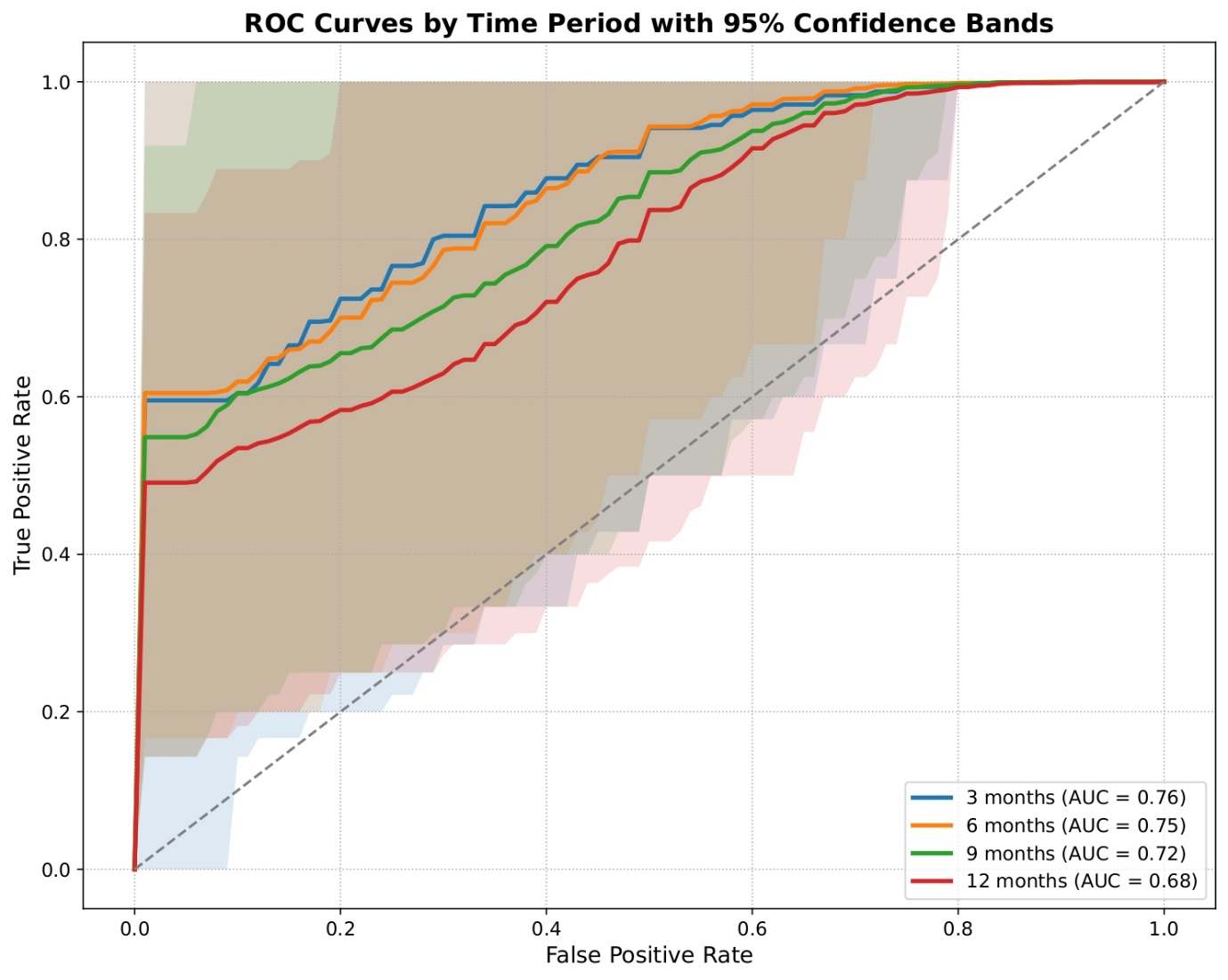


**Figure S2.** ROC curves for the XGBoost model on the testing set at 3, 6, 9, and 12 months before clinic visits. The XGBoost model uses ROC curves to identify flare versus non-flare events at 3, 6, 9, and 12 months before clinic appointments. The XGBoost model generated AUC values of 0.76 at 3 months, 0.75 at 6 months, 0.68 at 9 months, and 0.64 at 12 months before clinic visits. The predictive performance at 3 and 6 months is better because both achieved the highest AUC values among all time points.

S6 Calibration Plot


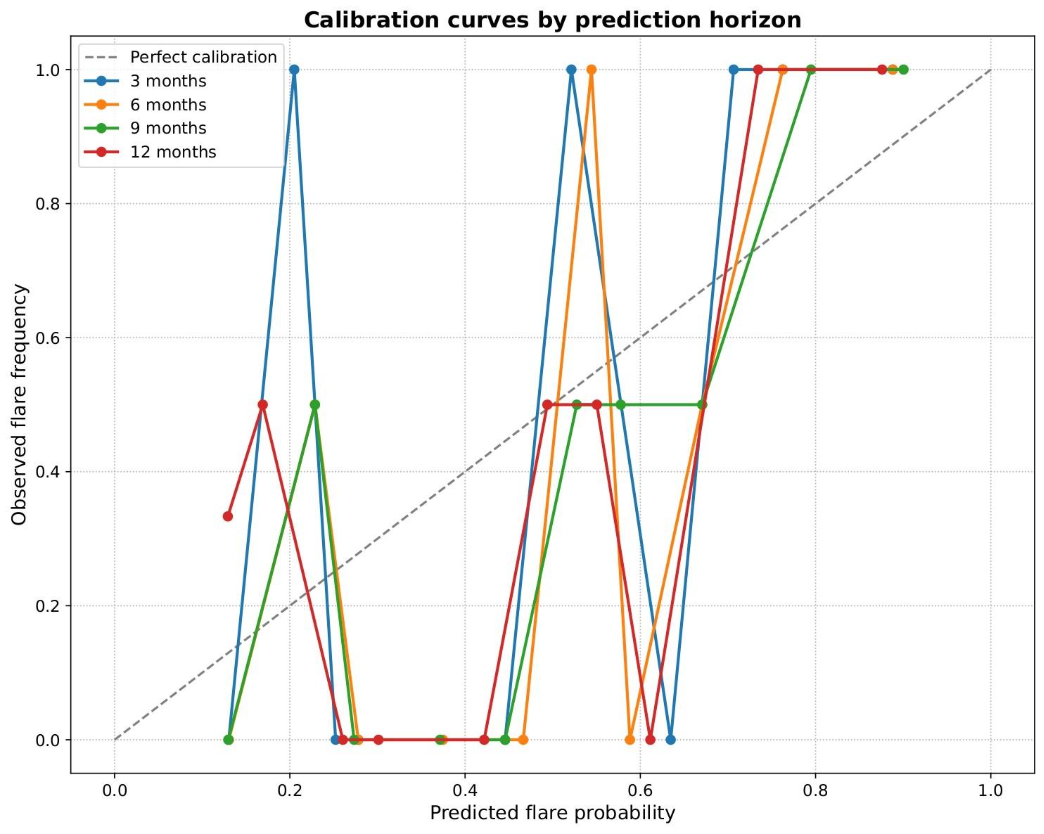


**Figure S3**. Calibration curves for LGBM predicted flare risk at 3, 6, 9, and 12 months.

Observed flare frequencies are plotted against mean predicted probabilities in deciles of predicted risk for each horizon. The dashed line represents perfect calibration. The model demonstrates good calibration across the low to moderate risk range, with mild deviations at higher predicted probabilities where sample sized are smaller.


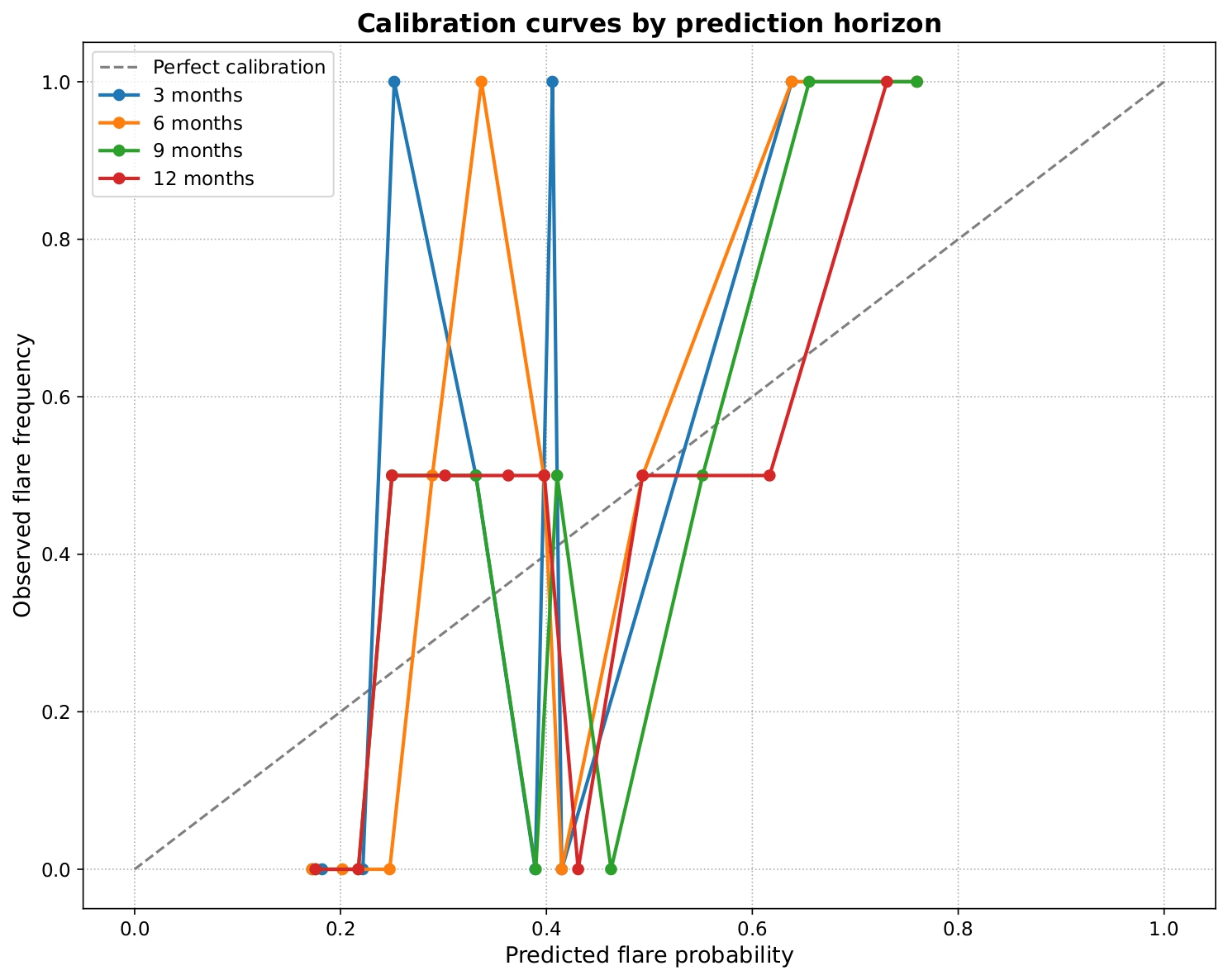


**Figure S4**. Calibration curves for XGBoost by prediction horizon (3,6,9, and 12months),

Calibration is generally well maintained across horizons, with increased variability and mild miscalibration at high predicted probabilities where sample sizes are limited.

## S7 Correlation Plot

We employed the Pearson correlation coefficient method on LGBM and XGBoost machine learning techniques on the training set and the testing set. The correlation was verified on the features to the target variable and the feature-to-feature correlation. It is explained in the following plots.


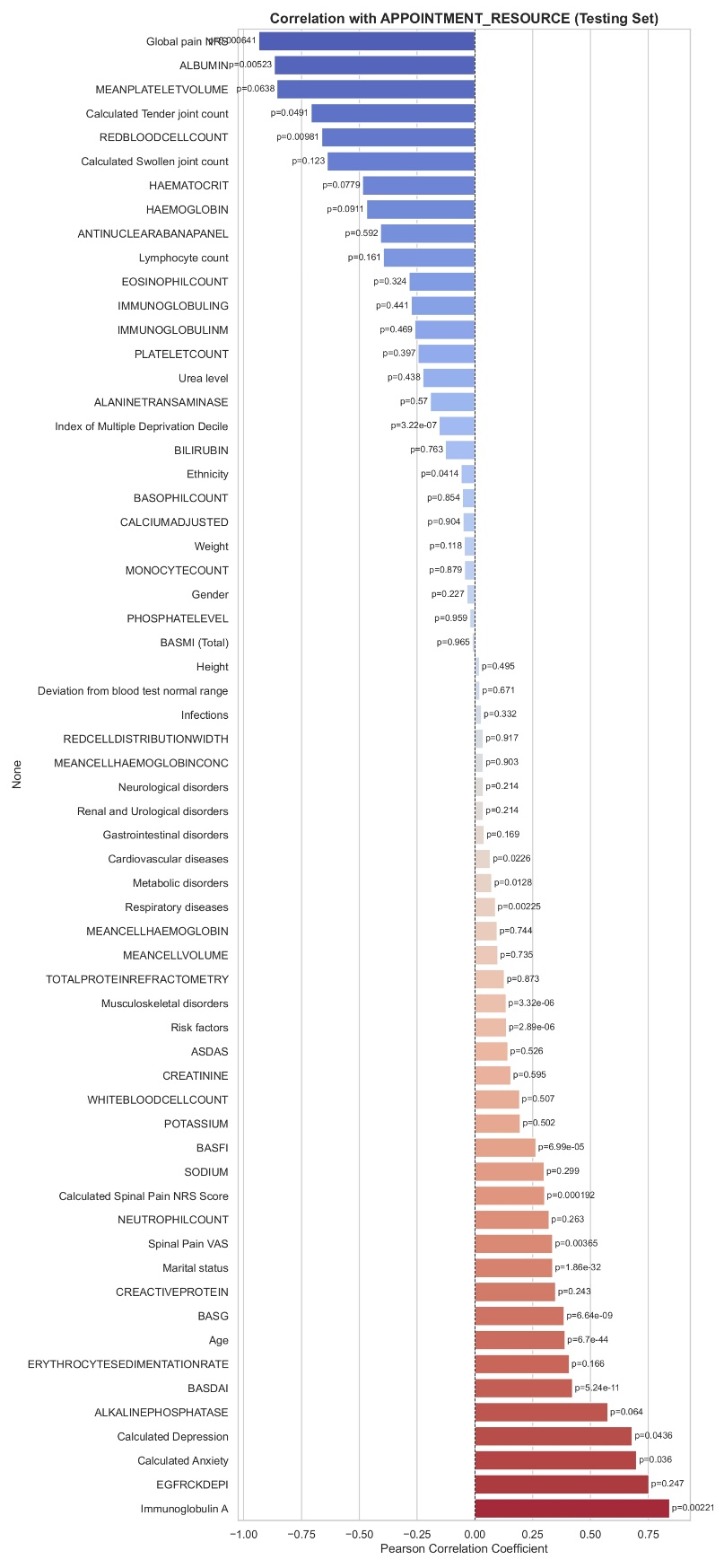

**Figure S5.** Pearson correlation between features and target variable (Appointment Resource) on testing set


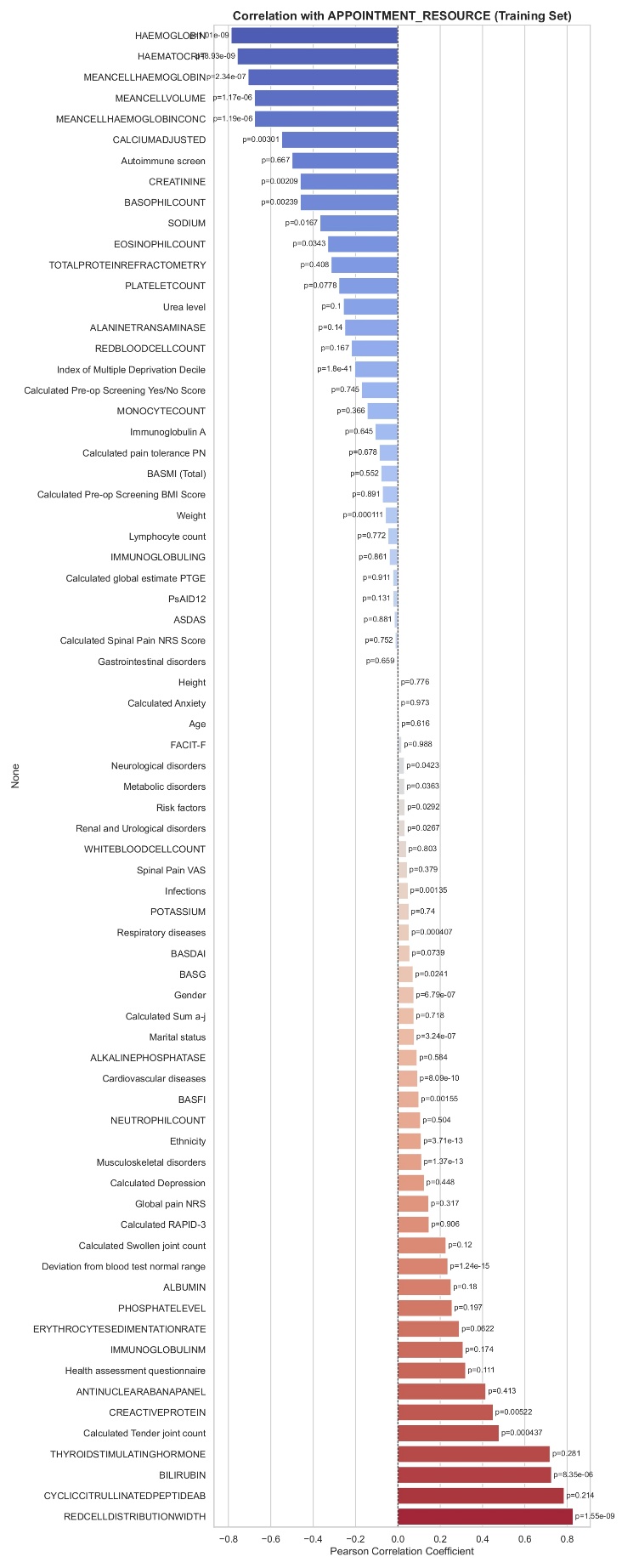


**Figure S6.** The Pearson correlation coefficients for LGBM on the training set between each input feature and the target variable (Appointment Resource) in the training set demonstrate the linear relationships. The analysis displays both positively and negatively correlated features with their respective p-values. The strength of the association with flare or non-flare outcomes increases as absolute correlation values increase.

### S7.1 Correlation analysis on training set

The table shows the correlation analysis on training set with variable category, variable, correlation coefficient and p-value.

| **Variable category** | **Variable** | **Correlation coefficient** | **P value** |
| --- | --- | --- | --- |
| Electronic Patient Reported Outcomes (ePROMs) | ASDAS | 0.014 | 9.02 × 10⁻¹ |
|  | **BASDAI** | **0.087** | **5.75 × 10⁻³** |
|  | **BASFI** | **0.128** | **4.54 × 10⁻⁵** |
|  | **BASG** | **0.093** | **3.57 × 10⁻³** |
|  | BASMI (Total) | -0.059 | 6.56 × 10⁻¹ |
|  | Calculated Anxiety | 0.174 | 2.70 × 10⁻¹ |
|  | Calculated Depression | 0.176 | 2.64 × 10⁻¹ |
|  | Calculated Likert Pain NRS Score | -0.027 | 8.52 × 10⁻¹ |
|  | Calculated Pre-op Screening BMI Score | -0.072 | 8.91 × 10⁻¹ |
|  | Calculated Pre-op Screening Yes/No Score | -0.171 | **7.45 × 10⁻¹** |
|  | Calculated PsAID-12 Score | 0.320 | 1.10 × 10⁻¹ |
|  | Calculated RAPID-3 | -0.939 | 2.22 × 10⁻¹ |
|  | Calculated Spinal Pain NRS Score | 0.025 | 5.34 × 10⁻¹ |
|  | Calculated Sum a-j | -0.365 | 7.94 × 10⁻² |
|  | Calculated Swollen joint count | 0.0510 | 7.36 × 10⁻¹ |
|  | Calculated Tender joint count | 0.191 | 1.88 × 10⁻¹ |
|  | **Calculated global estimate PTGE** | **-0.531** | **7.46 × 10⁻³** |
|  | **Calculated pain tolerance PN** | **-0.563** | **4.15 × 10⁻³** |
|  | FACIT-F | 0.018 | 9.88 × 10⁻¹ |
|  | PsAID12 | -0.022 | 1.10 × 10⁻¹ |
|  | Spinal Pain VAS | 0.066 | 1.95 × 10⁻¹ |
| HEIGHTWEIGHT | HEIGHTLENGTHMEASURED | -0.008 | 5.46 × 10⁻¹ |
|  | **WEIGHTMEASURED** | **-0.063** | **6.82 × 10⁻⁶** |
| BLOOD TEST | **Deviation from blood test normal range** | **0.130** | **2.95 × 10⁻⁶** |
|  | ALANINETRANSAMINASE | -0.257 | 1.08 × 10⁻¹ |
|  | ALBUMIN | 0.269 | 1.29 × 10⁻¹ |
|  | ALKALINEPHOSPHATASE | 0.062 | 6.89 × 10⁻¹ |
|  | ANTINUCLEARABANAPANEL | 0.440 | 3.22 × 10⁻¹ |
|  | **BASOPHILCOUNT** | **-0.429** | **2.57 × 10⁻³** |
|  | **BILIRUBIN** | **0.712** | **4.85 × 10⁻⁶** |
|  | **CALCIUMADJUSTED** | **-0.552** | **1.54 × 10⁻³** |
|  | CREACTIVEPROTEIN | 0.194 | 2.11 × 10⁻¹ |
|  | **CREATININE** | **-0.465** | **8.47 × 10⁻⁴** |
|  | CYCLICCITRULLINATEDPEPTIDEAB | 0.733 | 1.59 × 10⁻¹ |
|  | DSDNAELISA | -0.500 | 6.67 × 10⁻¹ |
|  | **EOSINOPHILCOUNT** | **-0.309** | **3.44 × 10⁻²** |
|  | ERYTHROCYTESEDIMENTATIONRATE | 0.183 | 2.18 × 10⁻¹ |
|  | **HAEMATOCRIT** | **-0.747** | **1.52 × 10⁻⁹** |
|  | **HAEMOGLOBIN** | **-0.772** | **1.96 × 10⁻¹⁰** |
|  | IMMUNOGLOBULINA | -0.140 | 5.03 × 10⁻¹ |
|  | IMMUNOGLOBULING | -0.018 | 9.29 × 10⁻¹ |
|  | IMMUNOGLOBULINM | 0.319 | 1.20 × 10⁻¹ |
|  | LYMPHOCYTECOUNT | -0.014 | 9.25 × 10⁻¹ |
|  | **MEANCELLHAEMOGLOBIN** | **-0.688** | **8.70 × 10⁻⁸** |
|  | **MEANCELLHAEMOGLOBINCONC** | **-0.651** | **7.20 × 10⁻⁷** |
|  | **MEANCELLVOLUME** | **-0.656** | **5.30 × 10⁻⁷** |
|  | MONOCYTECOUNT | -0.163 | 2.72 × 10⁻¹ |
|  | NEUTROPHILCOUNT | 0.040 | 7.87 × 10⁻¹ |
|  | PHOSPHATELEVEL | 0.290 | 1.20 × 10⁻¹ |
|  | PLATELETCOUNT | -0.272 | 6.42 × 10⁻² |
|  | POTASSIUM | -0.025 | 8.65 × 10⁻¹ |
|  | REDBLOODCELLCOUNT | -0.256 | 8.22 × 10⁻² |
|  | **REDCELLDISTRIBUTIONWIDTH** | **0.830** | **1.10 × 10⁻¹⁰** |
|  | **SODIUM** | **-0.342** | **1.72 × 10⁻²** |
|  | THYROIDSTIMULATINGHORMONE | 0.673 | 1.42 × 10⁻¹ |
|  | TOTALPROTEINREFRACTOMETRY | -0.246 | 4.92 × 10⁻¹ |
|  | **UREALEVEL** | **-0.286** | **4.86 × 10⁻²** |
|  | WHITEBLOODCELLCOUNT | -0.005 | 9.73 × 10⁻¹ |
| DEMOGRAPHICS | **Index of Multiple Deprivation Decile** | **-0.215** | **5.06 × 10⁻⁵³** |
|  | AGE_AT_APPOINTMENT_DATE | 0.019 | 1.60 × 10⁻¹ |
|  | **ETHNICITY** | **0.060** | **1.54 × 10⁻⁵** |
|  | **PERSON_GENDER_CODE_LOCAL** | **0.110** | **2.60 × 10⁻¹⁵** |
|  | **PERSON_MARITAL_STATUS_CODE_LOCAL** | **0.102** | **2.06 × 10⁻¹³** |
| COMORBIDITY | **Cardiovascular diseases** | **0.072** | **2.61 × 10⁻⁷** |
|  | Gastrointestinal disorders | 0.001 | 9.11 × 10⁻¹ |
|  | **Infections** | **0.041** | **2.94 × 10⁻³** |
|  | Metabolic disorders | 0.037 | 8.07 × 10⁻³ |
|  | **Musculoskeletal disorders** | **0.106** | **3.41 × 10⁻¹⁴** |
|  | Neurological disorders | 0.026 | 5.88 × 10⁻² |
|  | **Renal and Urological disorders** | **0.029** | **3.58 × 10⁻²** |
|  | **Respiratory diseases** | **0.056** | **5.46 × 10⁻⁵** |
|  | **Risk factors** | **0.033** | **1.80 × 10⁻²** |
| Drugs | **DRUGS_csDMARD** | **-0.094** | **1.81 × 10⁻¹¹** |
|  | DRUGS_tsDMARD | -0.016 | 2.43 × 10⁻¹ |
|  | **DRUGS_bDMARD** | **-0.124** | **6.26 × 10⁻¹⁹** |
|  | **DRUGS_Steroids** | **-0.034** | **1.44 × 10⁻²** |

**Table S2:** Correlation analysis on training set with category variable, variable, correlation coefficient and p-value.


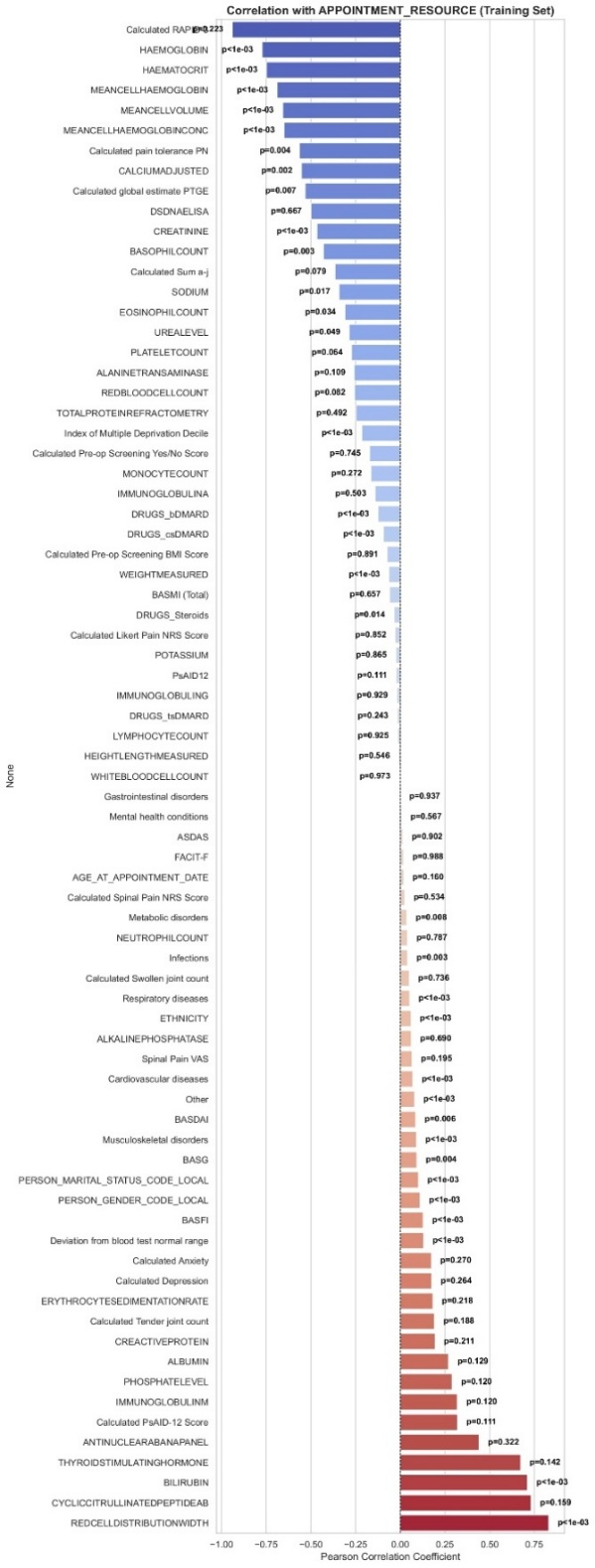


**Figure S7.** The Pearson correlation coefficients for XGBoost on the training set between each input feature and the target variable (Appointment Resource) in the training set demonstrate the linear relationships. The analysis displays both positively and negatively correlated features with their respective p-values. The strength of the association with flare or non-flare outcomes increases as the absolute correlation values increase.


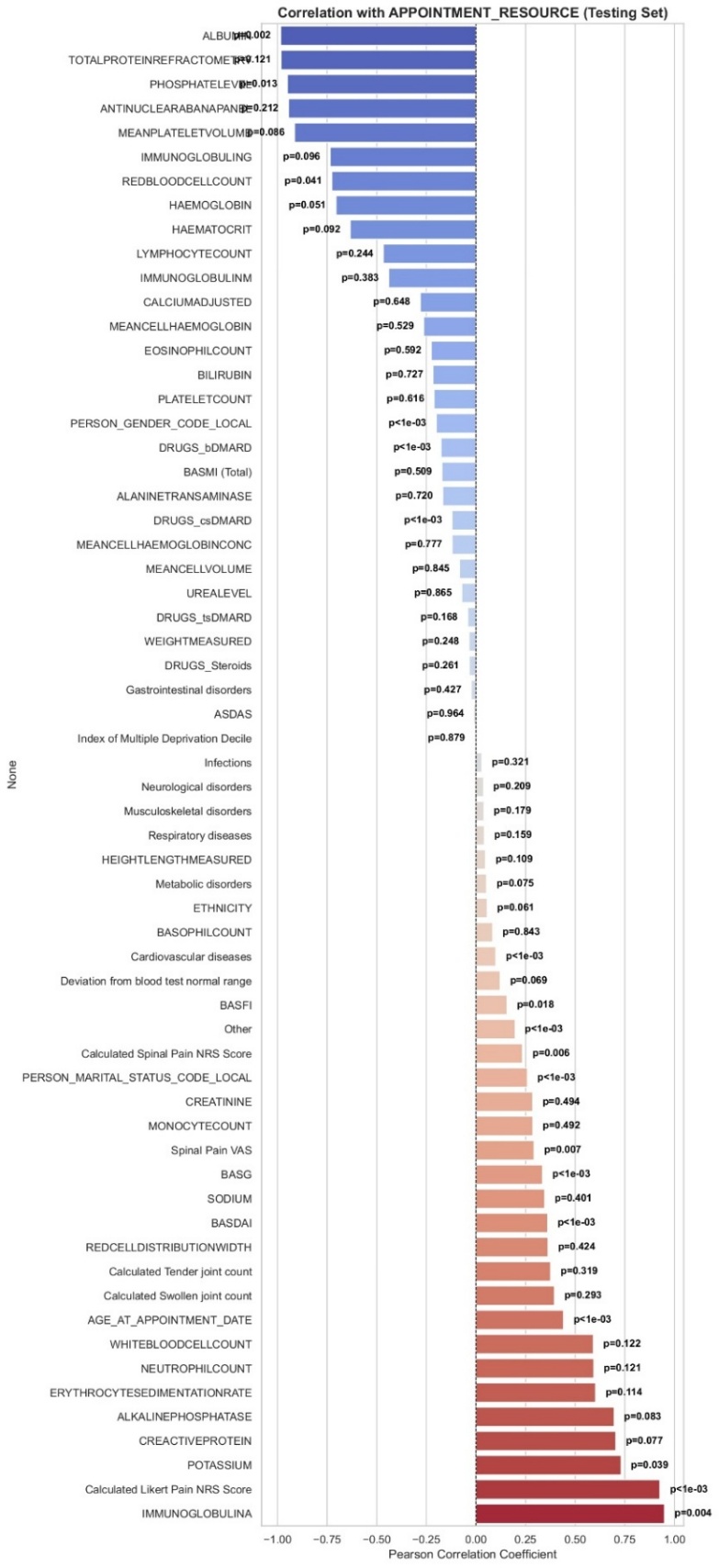


**Figure S8.** The Pearson correlation coefficients for XGBoost on the testing set between each input feature and the target variable (Appointment Resource) in the testing set demonstrate the linear relationships. The analysis displays both positively and negatively correlated features with their respective p-values. The strength of the association with flare or non-flare outcomes increases as the absolute correlation values increase.

## S8 Feature-to-feature correlation plot


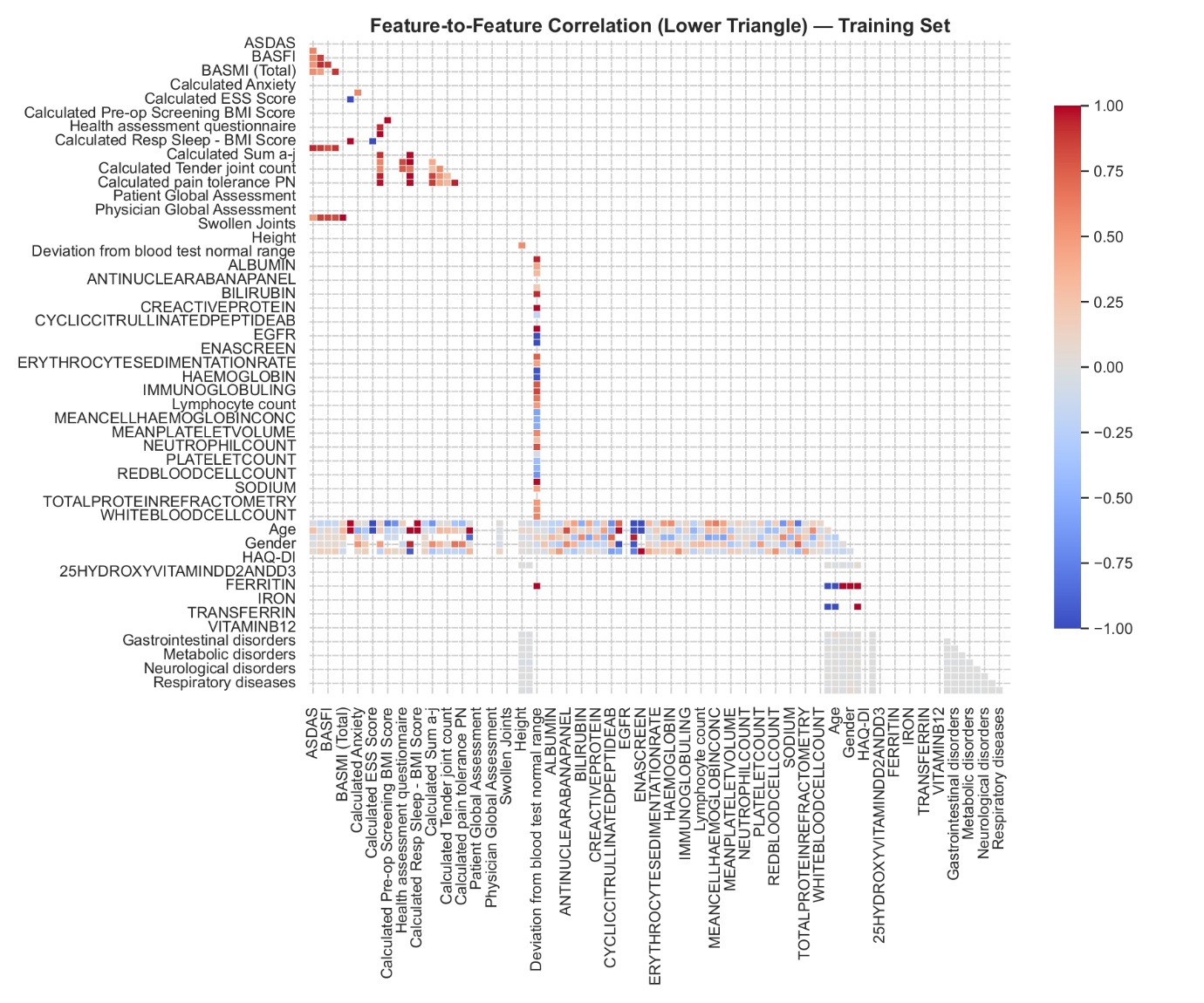


**Figure S9.** The plot displays the lower triangle of the Pearson correlation matrix of the LGBM model, which represents the features in the training set. The plot displays positive correlations with red colours while negative correlations use blue colours, and the intensity of these colours signifies the strength of the relationships. The analysis detects multicollinearity between features utilised by the LGBM model.


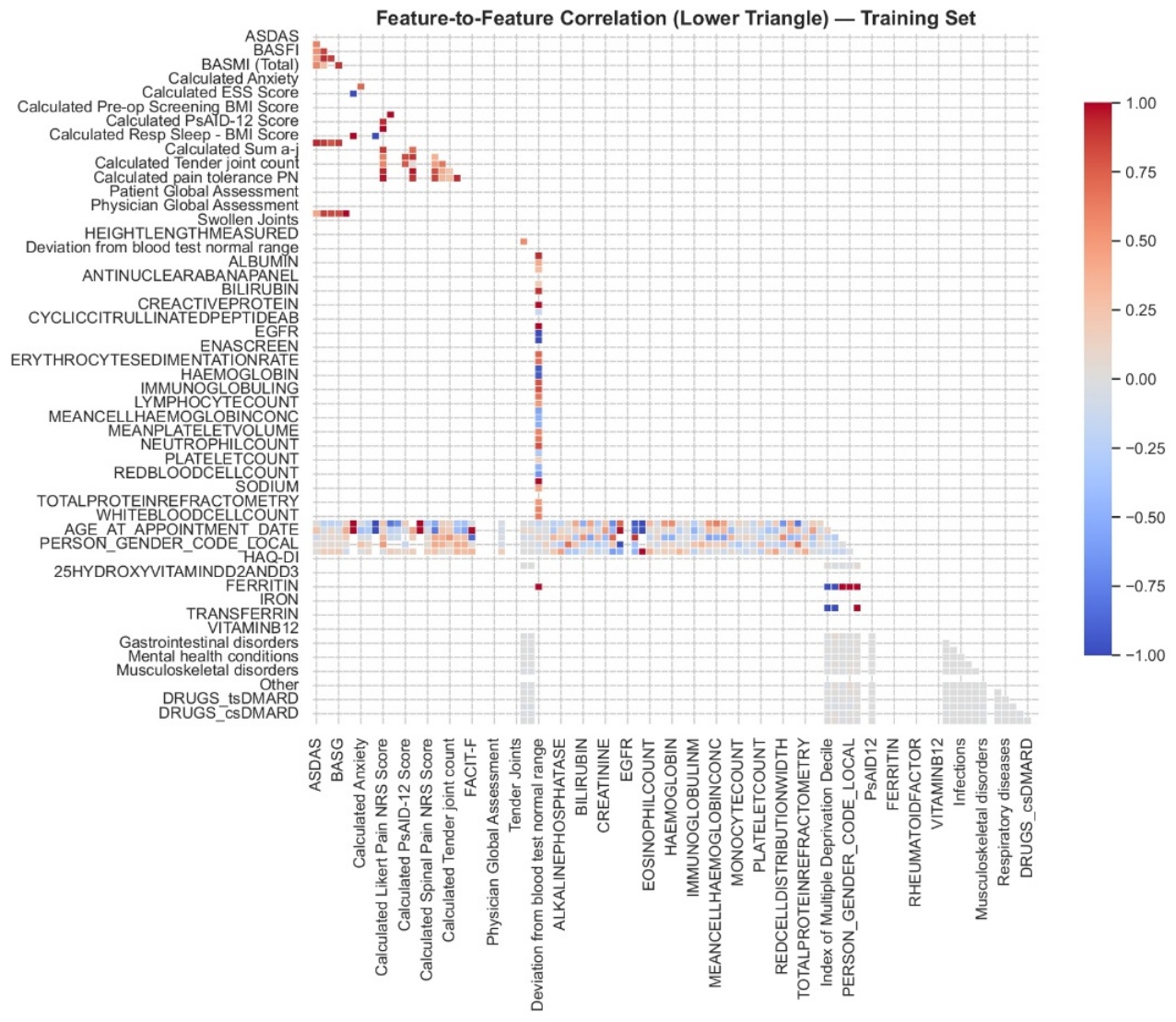


**Figure S10.** The plot displays the lower triangle of the Pearson correlation matrix of the XGBoost model, which represents the features in the training set. The plot displays positive correlations with red colours while negative correlations use blue colours, and the intensity of these colours signifies the strength of the relationships. The analysis detects multicollinearity between features utilised by the XGBoost model.


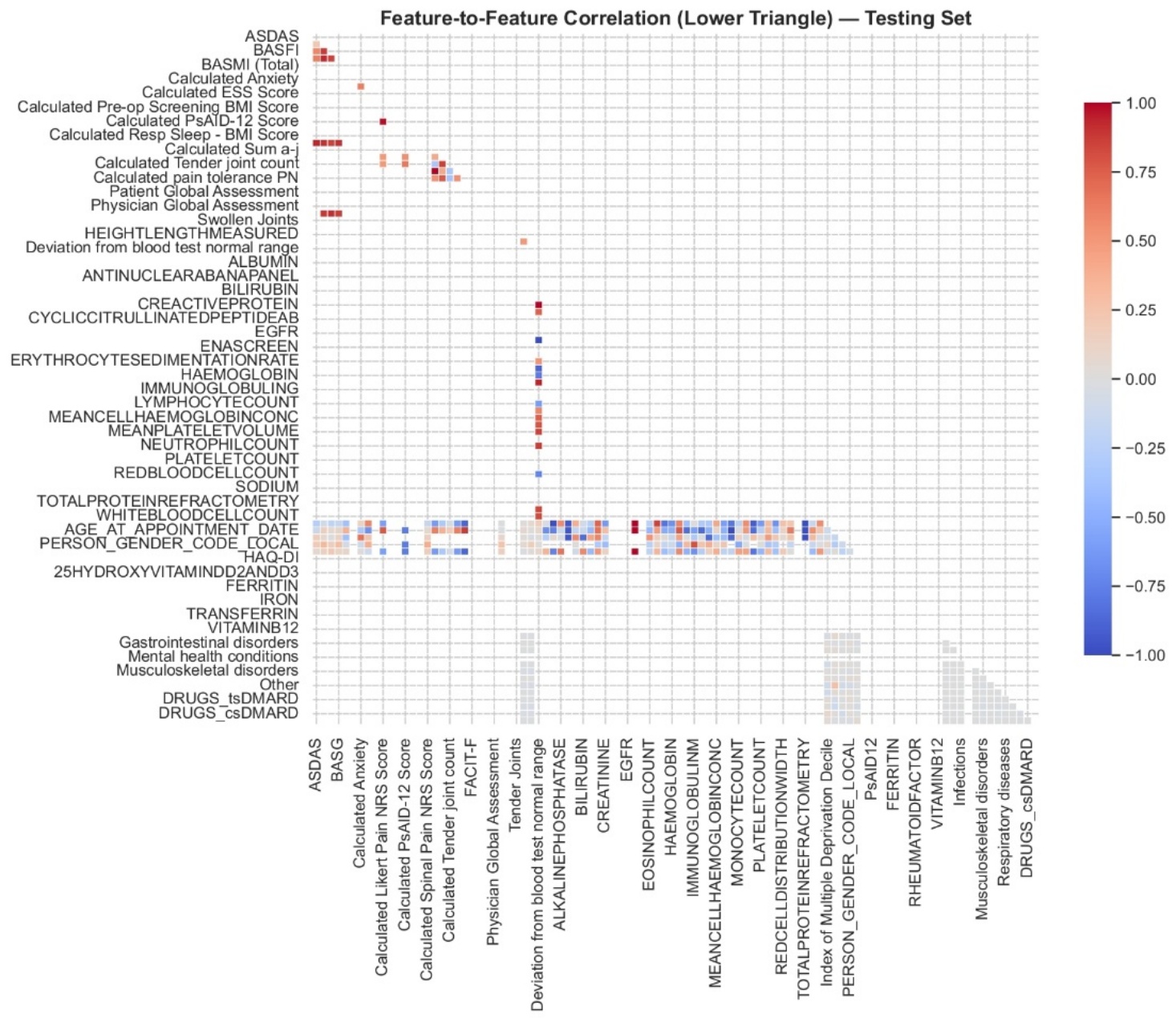


**Figure S11.** The plot displays the lower triangle of the Pearson correlation matrix of the XGBoost model, which represents the features in the testing set. The plot displays positive correlations with red colours while negative correlations use blue colours, and the intensity of these colours signifies the strength of the relationships. The analysis detects multicollinearity between features utilised by the XGBoost model.

## S9 Chi-square analysis on training set

| **Categorical feature** | **Ethinicity label** | **Chi-squared (X²)** | **Mean target** | **Count** | **P-value** |
| --- | --- | --- | --- | --- | --- |
| Ethnicity | Asian | X²(3) = 135.85 | 0.606 | 310 | 2.97×10⁻²⁹ |
|  | Mixed |  | 0.000 | 8 |  |
|  | Other |  | 0.250 | 810 |  |
|  | White |  | 0.321 | 3829 |  |
|  | **Marital status label** |  | **Mean target** | **Count** | **P-value** |
| Marital status | Divorced | X²(6) = 440.78 | 0.152 | 59 | 4.73×10⁻⁹² |
|  | Married |  | 0.446 | 1861 |  |
|  | Separated |  | 0.000 | 46 |  |
|  | Partnership |  | 1.000 | 5 |  |
|  | Single |  | 0.400 | 1293 |  |
|  | Widowed |  | 1.000 | 3 |  |
|  | **Gender label** |  | **Mean target** | **Count** | **P-value** |
| Gender | Male | X²(1) = 16.30 | 0.305 | 3142 | 5.42×10⁻⁵ |
|  | Female |  | 0.361 | 1826 |  |

**Table S3:** Chi-square test of independence of categorical variables in the training set. The table shows the mean of the dependent variable (1 = flare and 0 = no flare), the count of observations per category, and the p-value of the Chi-square test. The results are shown for three categorical predictors: ethnicity, marital status, and gender.  There was significant association between the dependent variable and each feature including ethnicity (X²(3) = 135.85, p = 2.97×10⁻²⁹), marital status (X²(6) = 440.78, p = 4.73×10⁻⁹²), and gender (X²(1) = 16.30, p = 5.42×10⁻⁵). These results suggest a meaningful association between these features and flare outcome in the training dataset.

## S10 SHAP Plot of variable importance for flare prediction


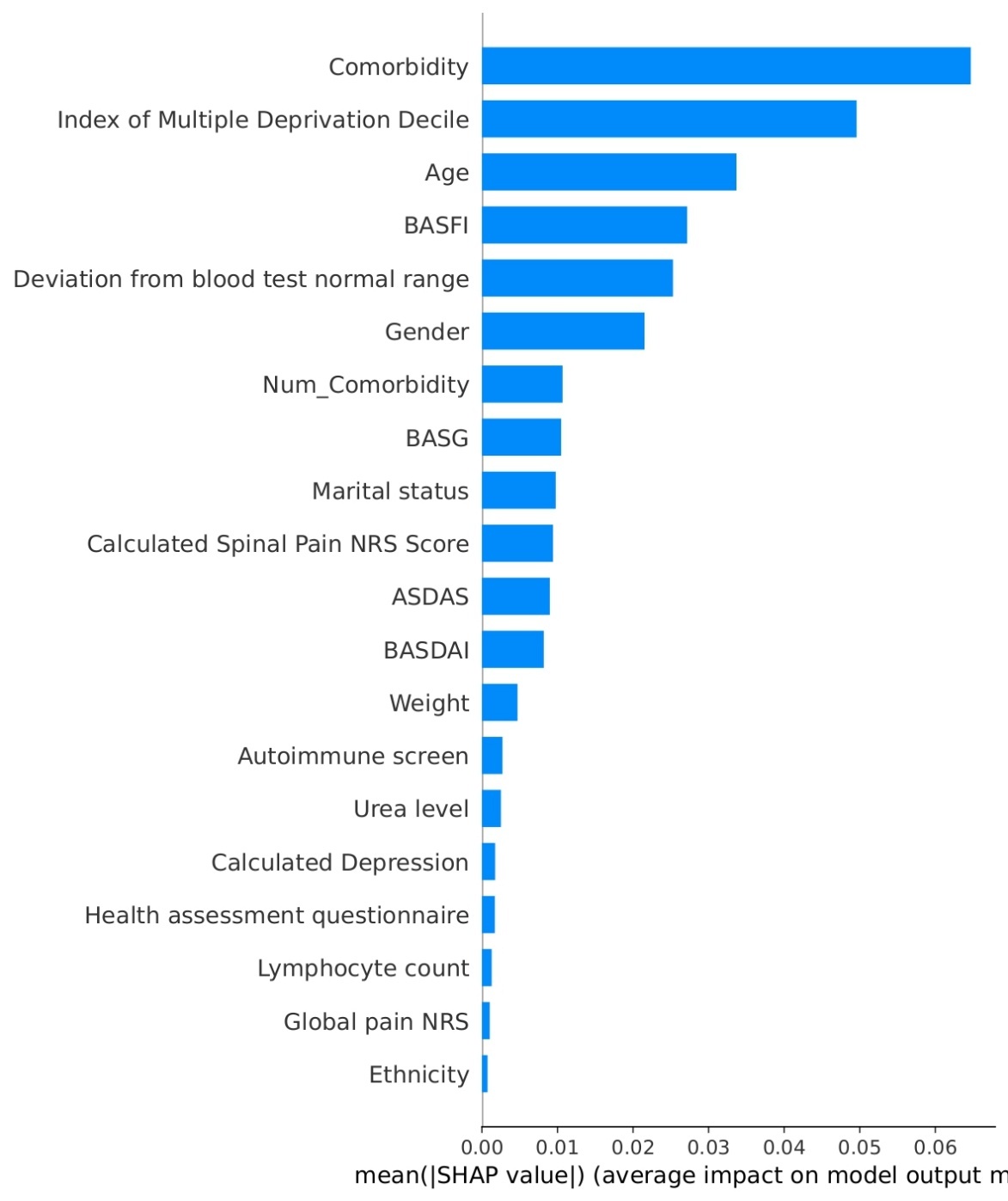


**Figure S12.** The diagram illustrates how the LGBM model predictions on the testing set are explained by the average SHAP values of different features. The comorbidity alongside Index of Multiple Deprivation Decile and age demonstrated the highest influence on flare prediction. Features with greater mean SHAP values play a more significant role in forecasting flare versus non-flare results.


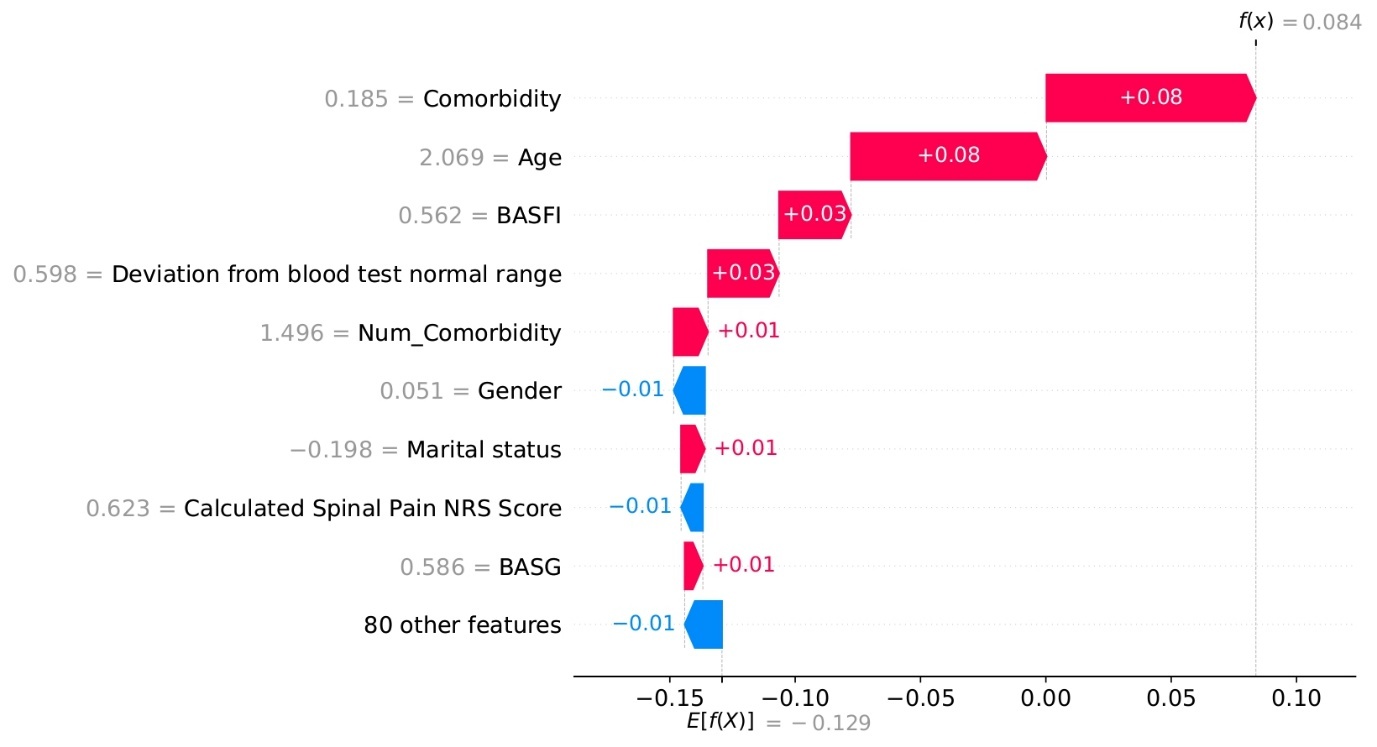


**Figure S13.** The SHAP waterfall plot demonstrates the individual feature contributions towards LGBM prediction from the test dataset. The prediction of flare risk increased because of comorbidity, age and BASFI, while it decreased due to Musculoskeletal disorders. All features moved the model output from its baseline value of -0.129 to reach the final predicted probability of 0.084.


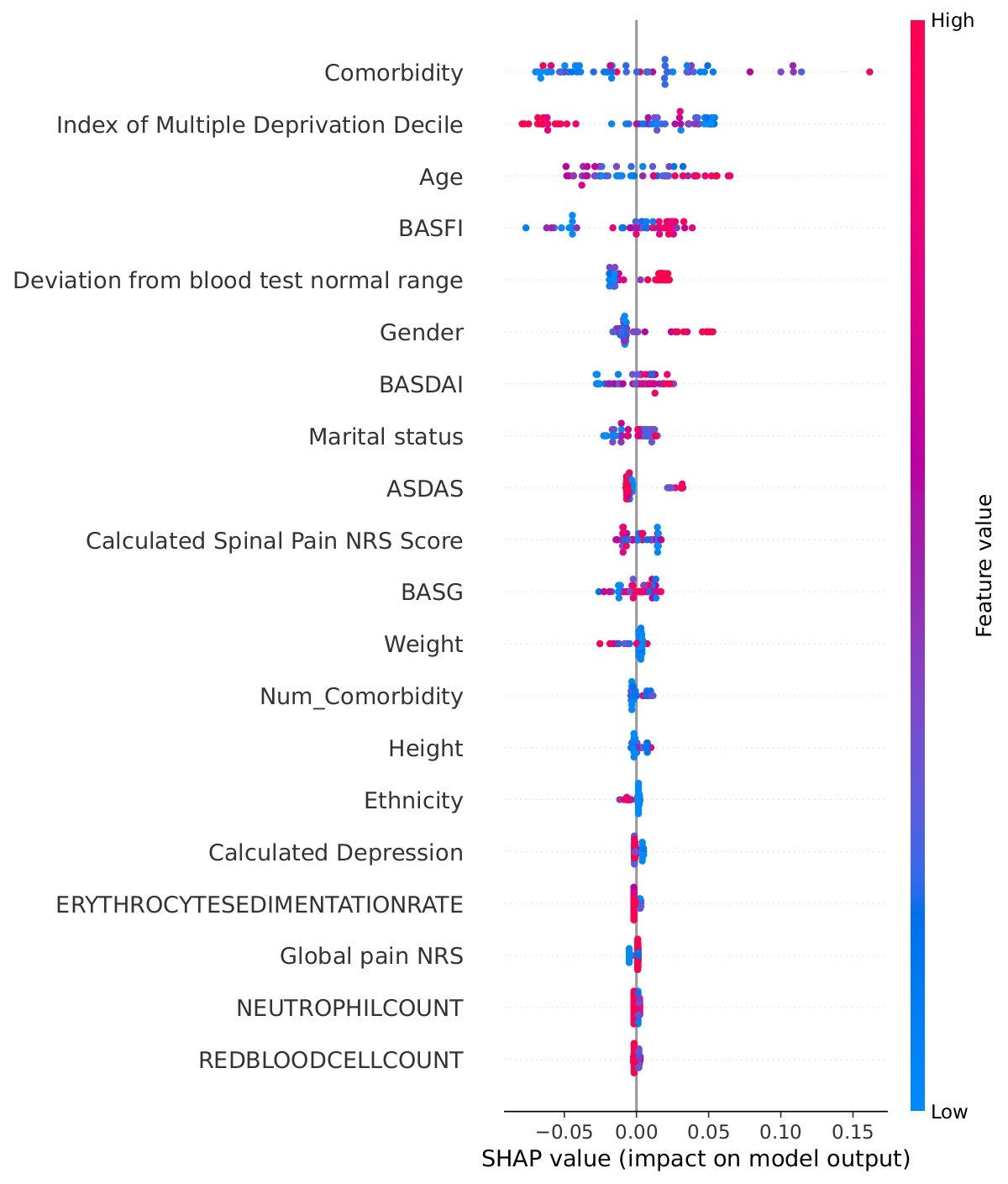


**Figure S14.** The SHAP dot plot demonstrates how each feature affects XGBoost model predictions when applied to the testing set. The colour of each dot reflects the feature value, where blue denotes low and red denotes high values, while its position displays the effect size for each patient. The comorbidity, Index of Multiple Deprivation Decile, age features demonstrated the most decisive influence on the model output.


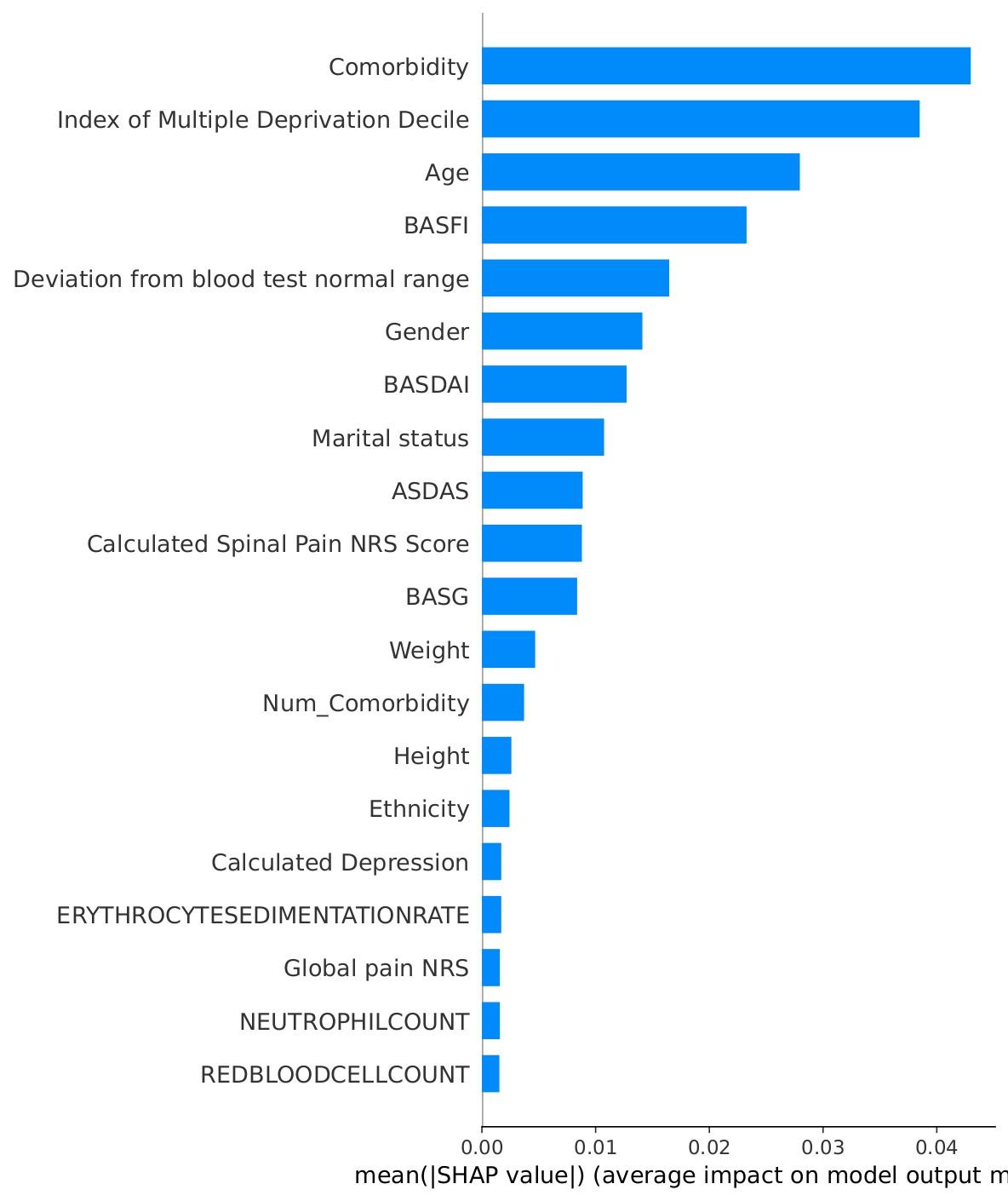


**Figure S15.** The diagram illustrates how the average SHAP values of different features explain the XGBoost model predictions on the testing set. The comorbidity alongside Index of Multiple Deprivation Decile and age demonstrated the highest influence of flare prediction. Features with greater mean SHAP values play a more significant role in forecasting flare versus non-flare results.


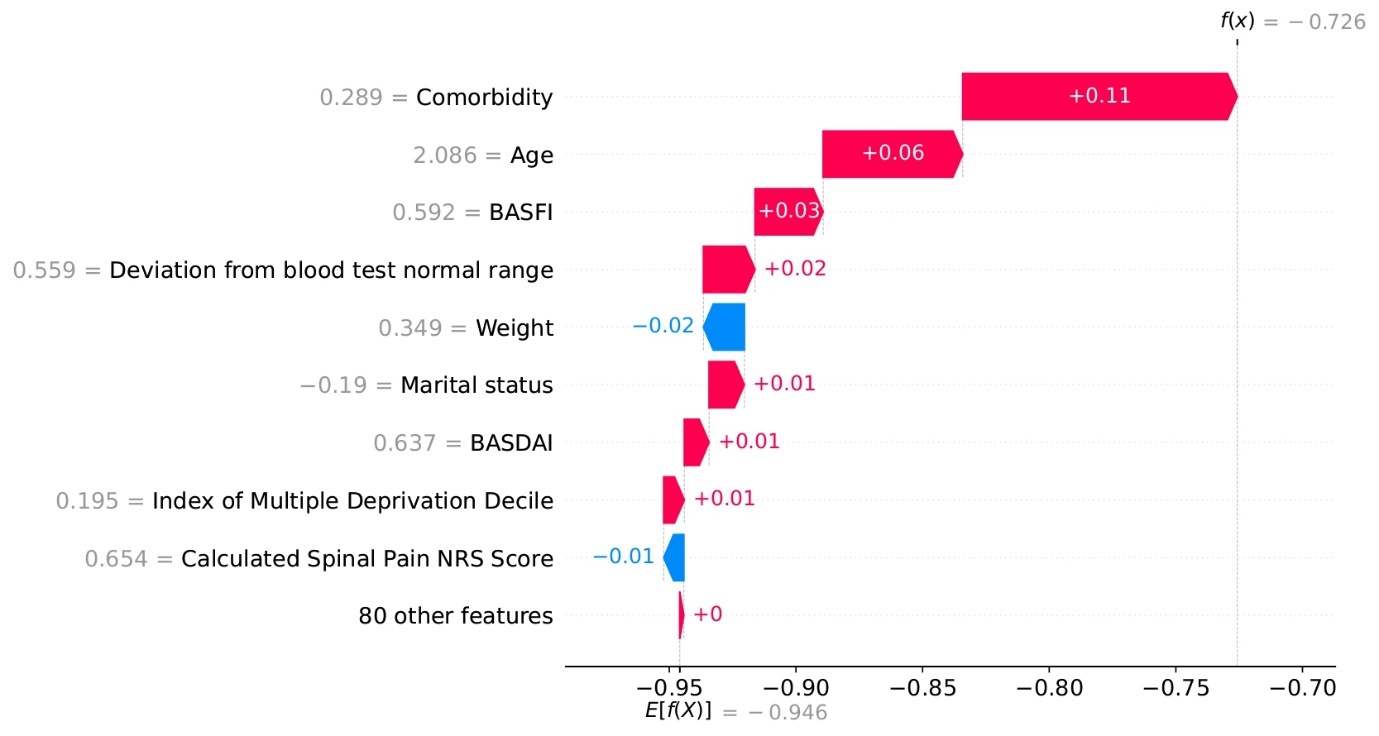


**Figure S16.** The SHAP waterfall plot displays the impact of each feature on the final prediction of an XGBoost model as it applies to test data.

The prediction of flare risk rose with comorbidity alongside age, and BASFI while BASFI and Spinal Pain NRS Score caused it to drop. All feature contributions together changed the model prediction from its expected value of –0.946 to a final output of –0.726.

## S11 Individual patient SHAP bar plot

Our research produced individual SHAP bar plots to show how the machine learning model predicted the risk of flare at different time intervals (3, 6, 9, and 12 months) before their scheduled clinic visits. Following the training of our LGBM and XGBoost model, we applied SHAP analysis to determine which patient features, like blood test results, pain scores and medication usage, most affected flare prediction accuracy. We developed bar charts to display the top 20 features that influenced flare predictions for individual patients and indicated if each feature raised or lowered flare risk. The plot explicitly showed each patient's predicted likelihood of experiencing or not experiencing a flare. The personalised plots reveal the decision-making process of the model throughout various time points, which aids clinicians in understanding the rationale behind each prediction and the model's reliability. The methodology produces clinically relevant predictions which may lead to more individualised patient treatment plans. In the following, we have provided some individual patient Shap bar plots for flare and non-flare for the LGBM and XGBoost models before attending the clinic for periods 3, 6, 9, and 12 months.

- LGBM (Flare Patients)


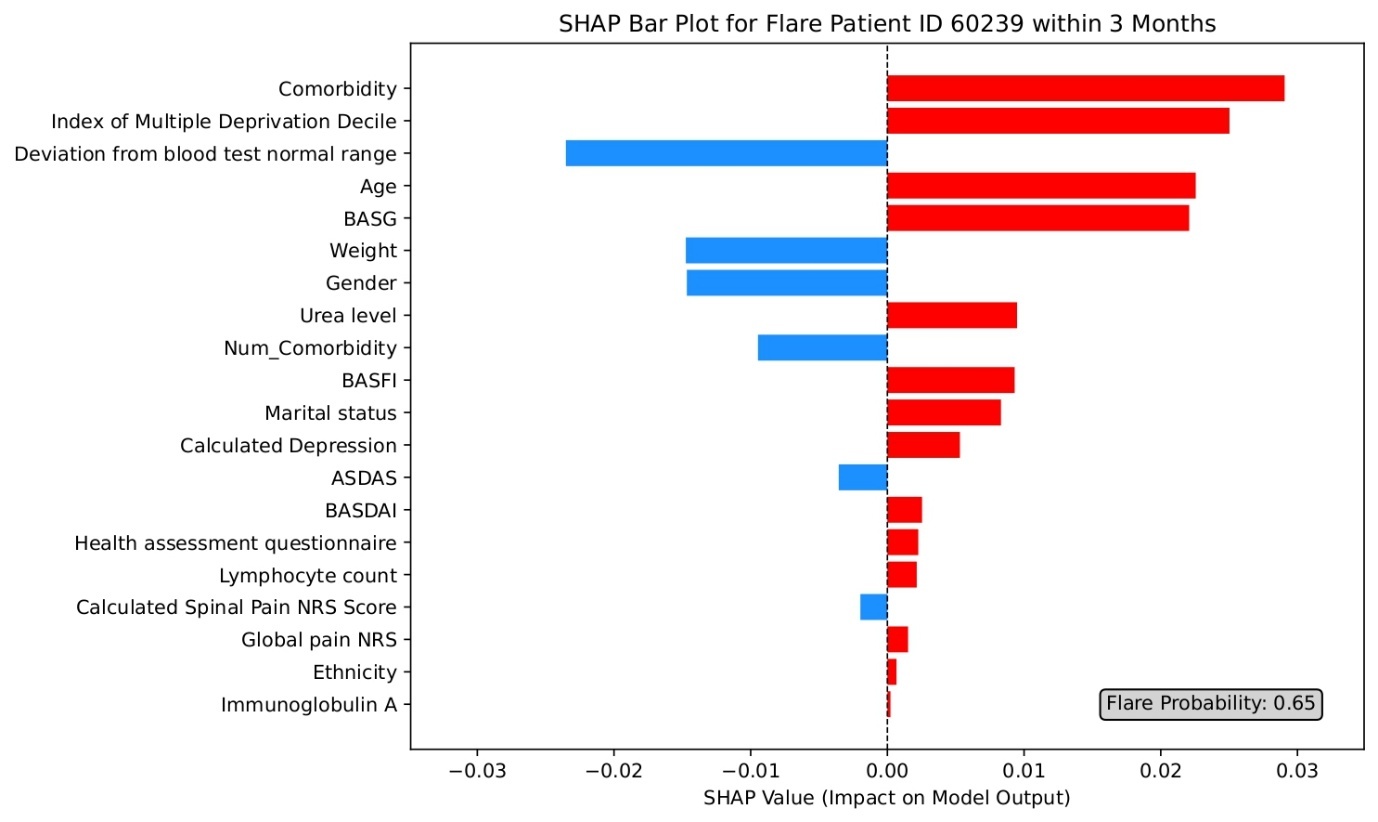


**Figure S17.** The SHAP bar plot displays which main features impacted the model's prediction of flare in the next three months following their previous clinic visit for Patient ID 60239. The model outputted a high flare likelihood score of 0.65, which was primarily influenced by comorbidity and Index of Multiple Deprivation Decile among other features.


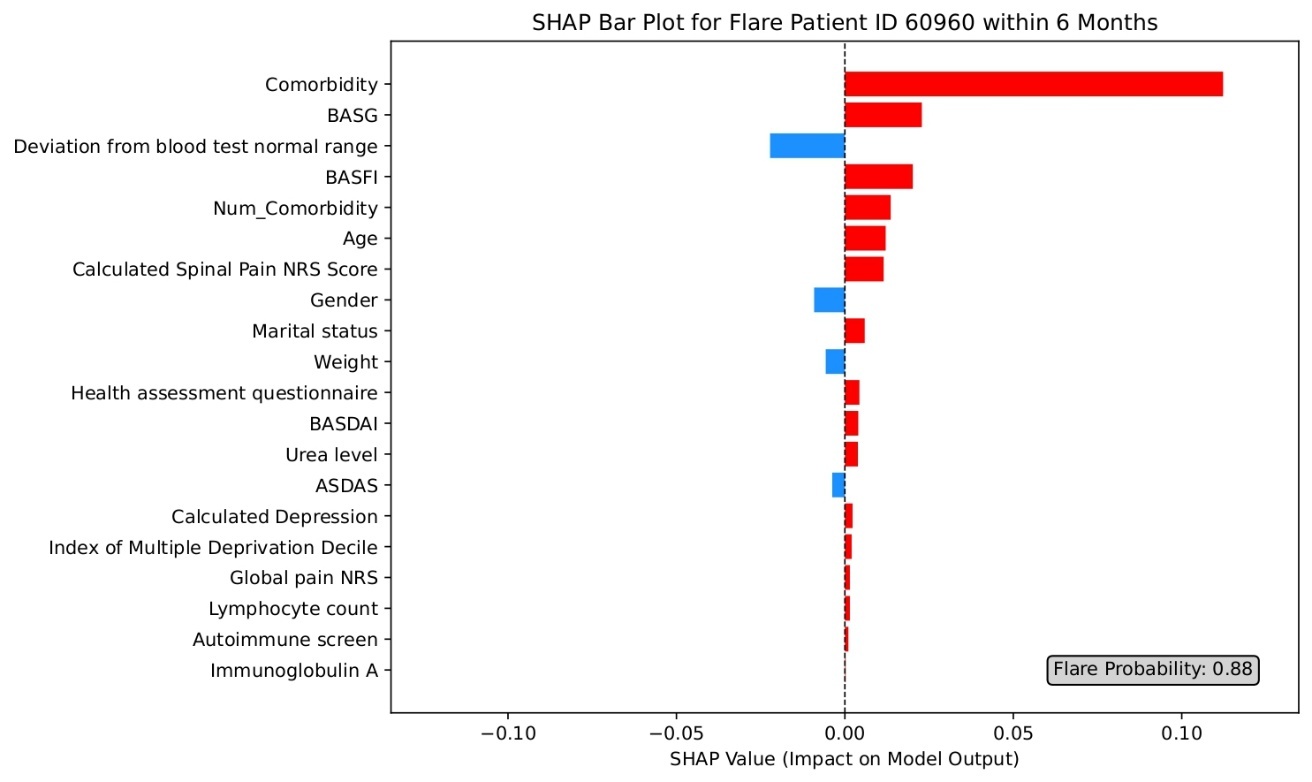


**Figure S18.** The SHAP bar plot demonstrates which features most affected the flare prediction for Patient ID 60960 in the next 6 months. According to the model, the flare prediction reached a high probability of 0.88 while comorbidity, BASG, and BASFI emerged as the top contributing factors.


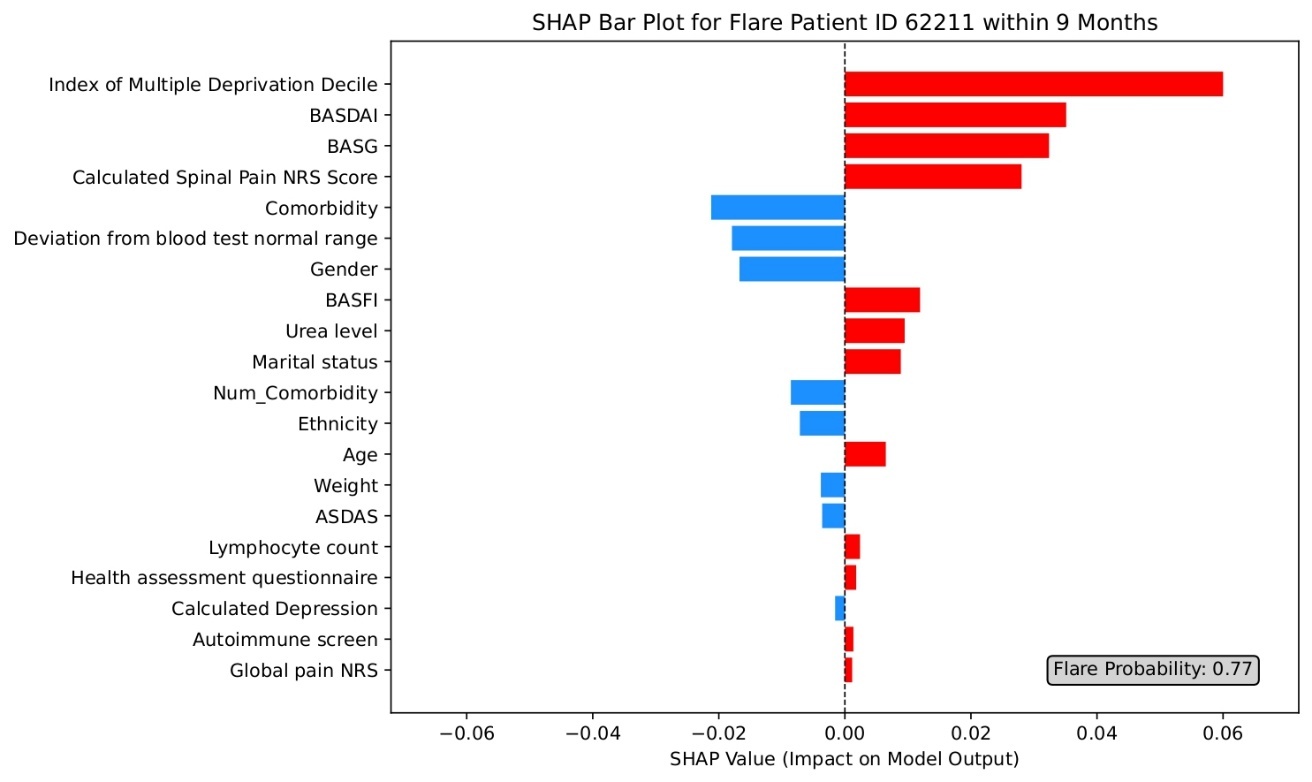


**Figure S19.** The SHAP bar plot illustrates which factors most impact the flare predictions for Patient ID 62211 in the next nine months. The model predicted a 0.77 flare probability with Index of Multiple Deprivation Decile, BASDAI, BASG and Calculated Spinal Pain NRS score as the strongest positive influencers.

- LGBM (Nonflare Patients)


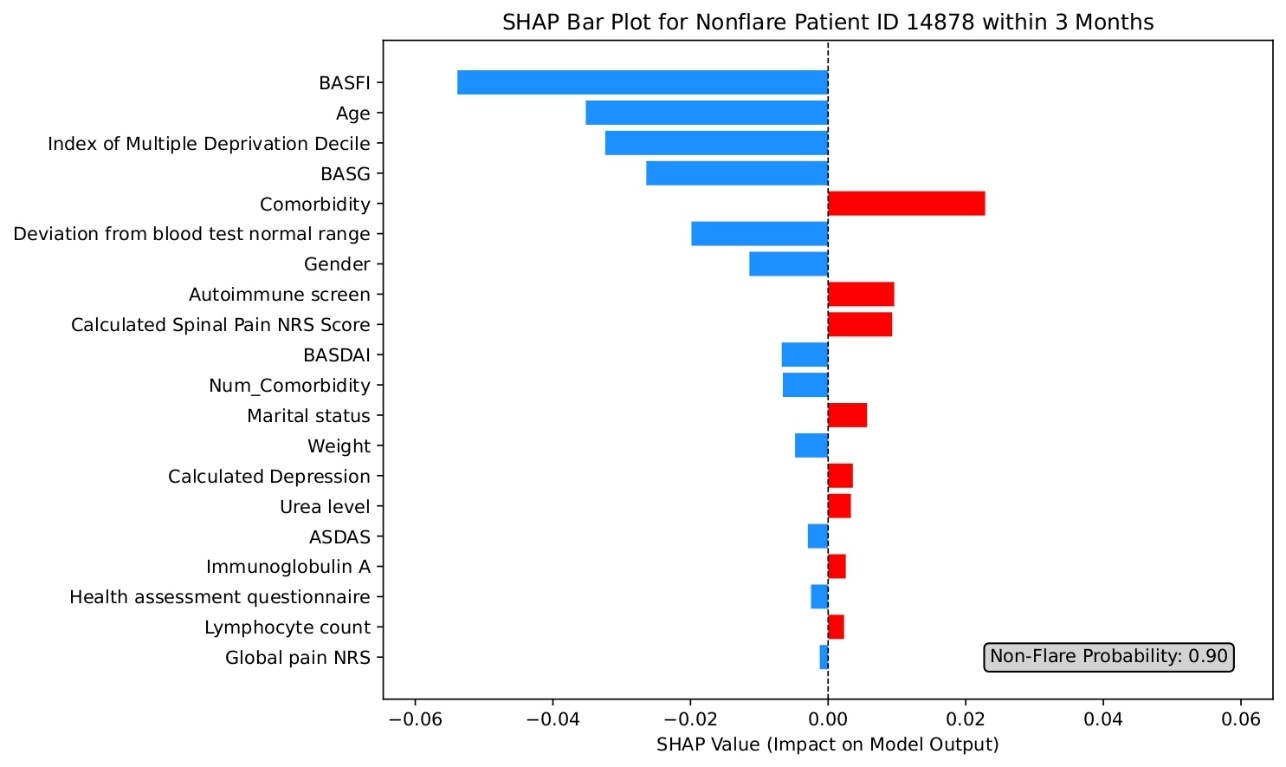


**Figure S20.** The SHAP bar plot identifies the primary factors that affect the forecast for Patient ID 14878 not having a flare event in the next three months. The prediction model calculated a non-flare probability of 0.90, which resulted primarily from factors such as BASFI, Age, Index of Multiple Deprivation Decile, BASG and Comorbidity.


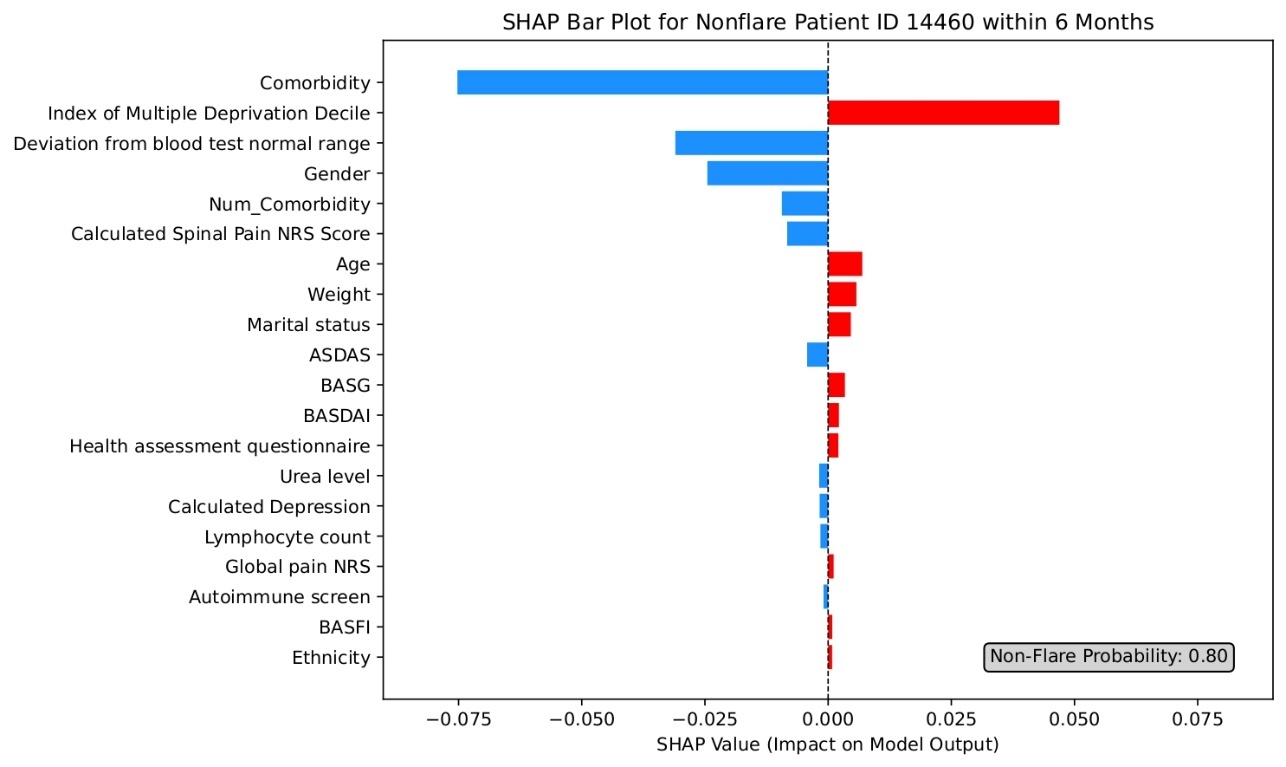


**Figure S21.** The SHAP bar plot reveals the principal features that lead to the forecast of a non-flare outcome for Patient ID 14460 in the next six months. The model predicted a 0.80 chance of not experiencing a flare-up, with the Comorbidity and Index of Multiple Deprivation Decile as the primary factors influencing this decision.


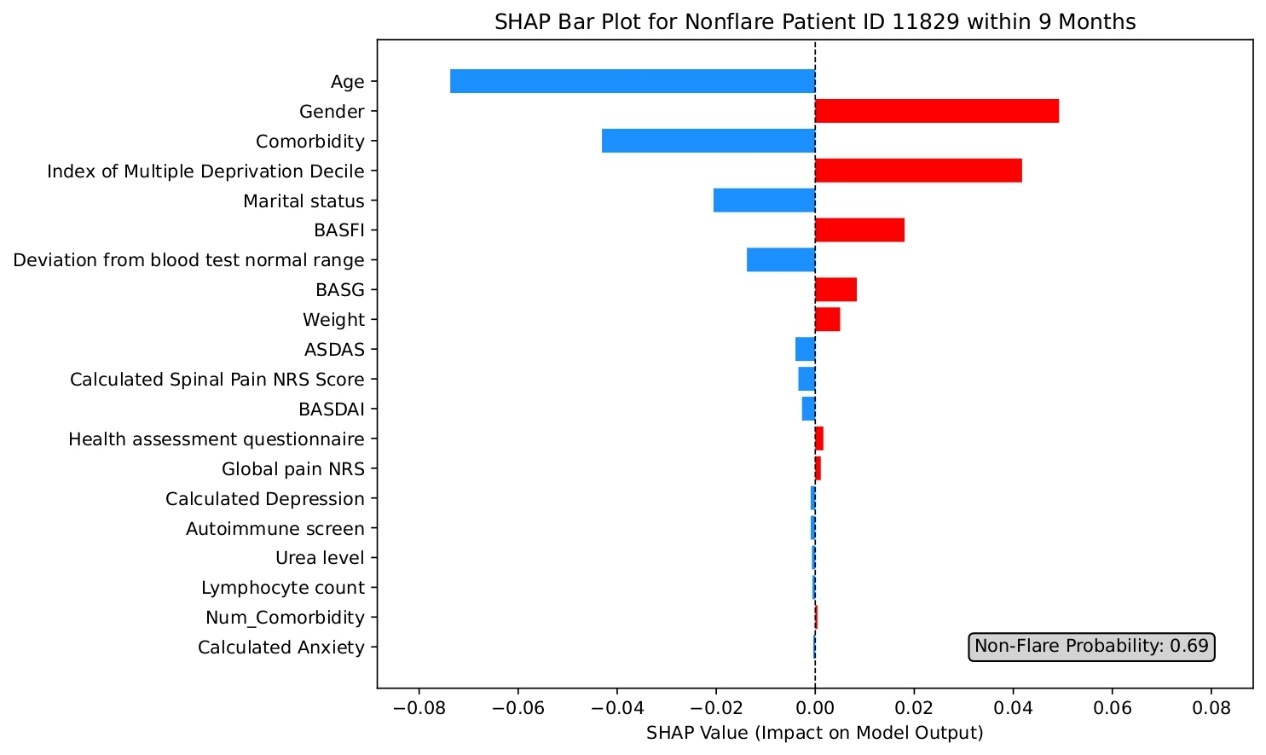


**Figure S22.** The SHAP bar plot illustrates the key features determining the non-flare prediction for Patient ID 11829 for 9 months. The model returned a 0.69 non-flare prediction probability, where age, gender, comorbidity, and Index of Multiple Deprivation Decile, emerged as the primary influencing factors.


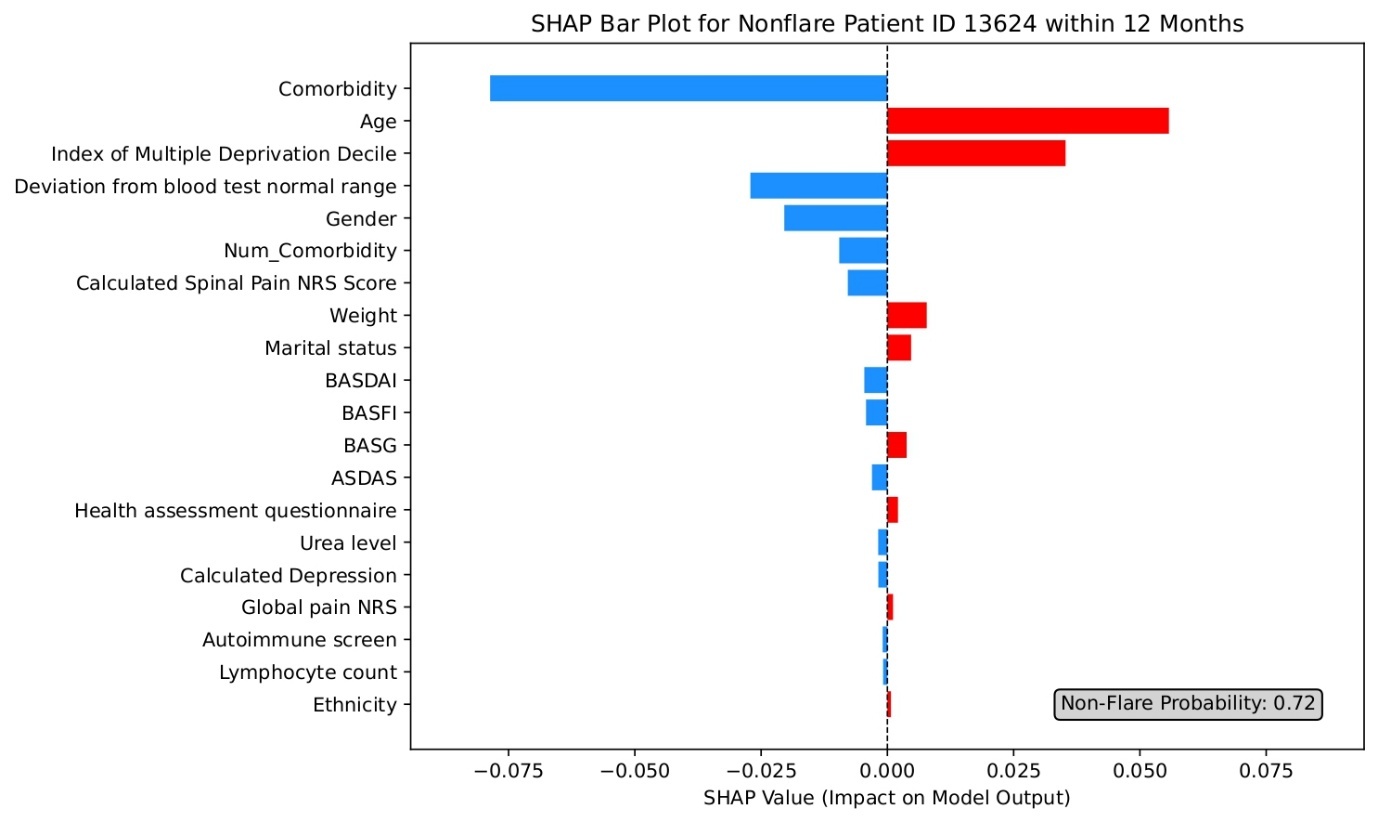


**Figure S23.** The SHAP bar plot reveals the principal factors affecting the non-flare prediction for Patient ID 13624 for 12 months. The model gave a non-flare chance of 0.72, which relied heavily on Comorbidity, Age, Index of Multiple Deprivation Decile and Deviation from blood test normal range.

XGBoost (Flare Patients)


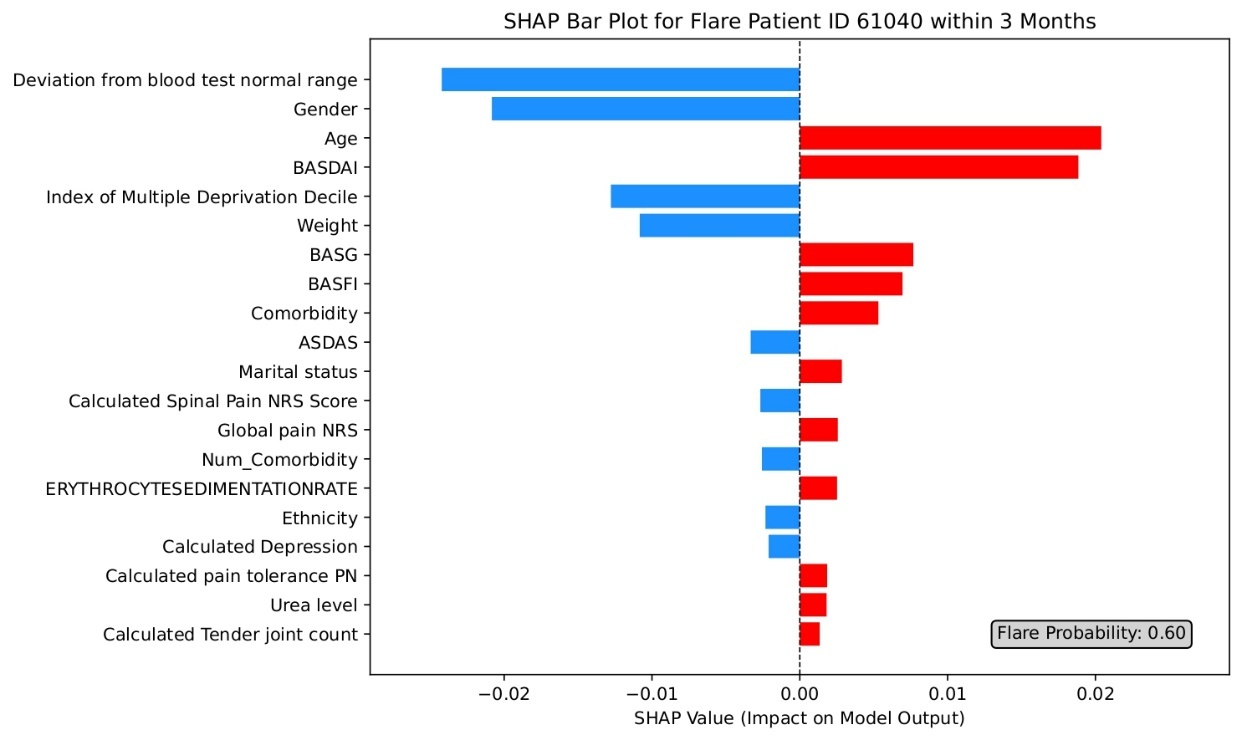


**Figure S24.** The SHAP bar plot illustrates the main features responsible for predicting a flare within 3 months for Patient ID 61040 and shows a predicted flare probability of 0.60. Deviation from blood test normal range, gender, age, BASDAI and Index of Multiple Deprivation Decile, emerged as the most significant factors that determine flare risk.


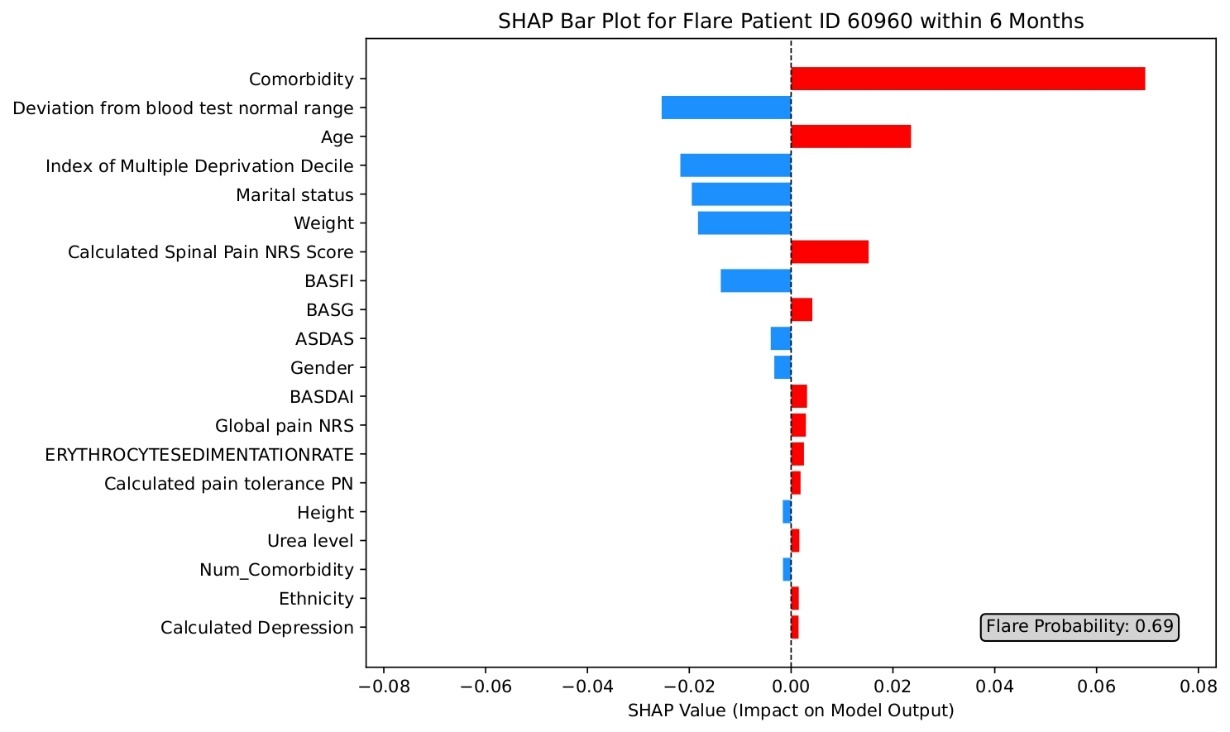


**Figure S25.** The SHAP bar plot identifies the main factors affecting the prediction of a flare for Patient ID 60960 within 6 months of next clinic while showing a predicted flare probability of 0.69. The Comorbidity, Deviation from blood test normal range, age, and Index of Multiple Deprivation Decile, emerged as the primary factors that contribute to flare risk.

- XGBoost (Nonflare Patients)


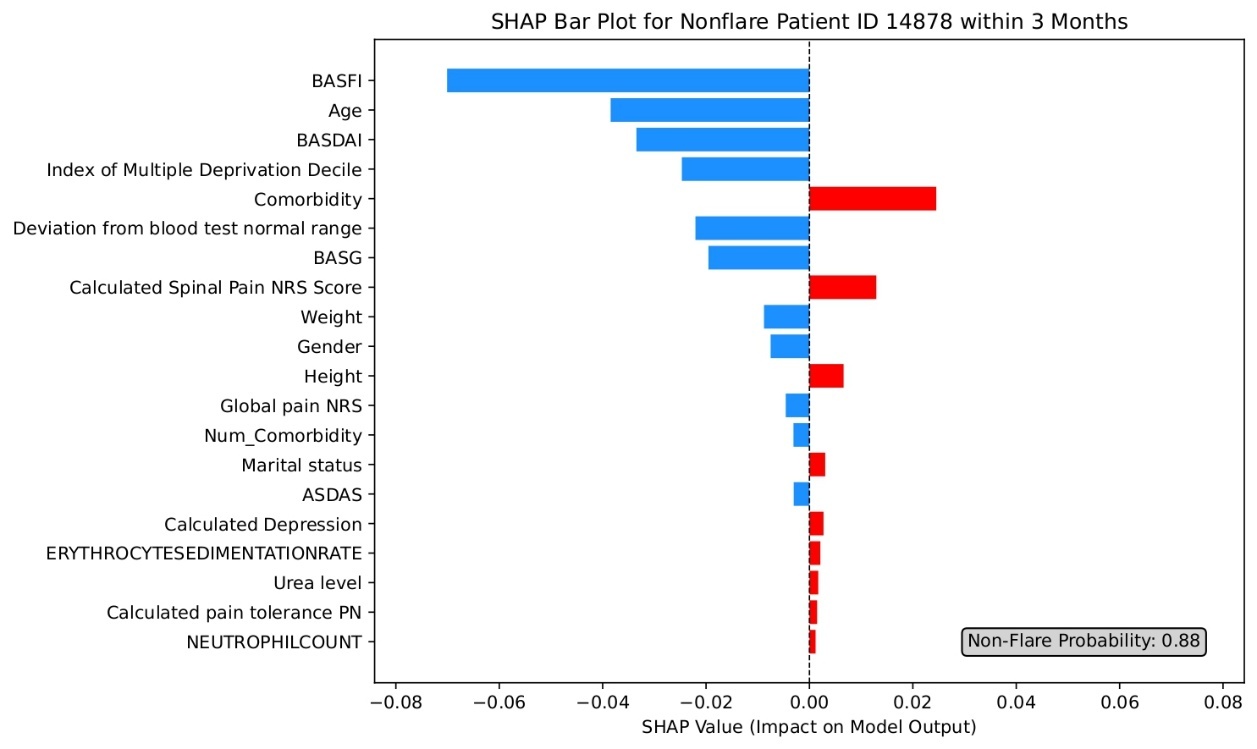


**Figure S26.** The SHAP bar plot reveals how different factors impact the prediction of no flare for Patient ID 14878 within three months of next clinic visit, with a calculated probability of non-flare standing at 0.88. BASFI, Age, BASDAI, Index of Multiple Deprivation Decile and Comorbidity, emerged as the primary features that sustained a non-flare outcome.


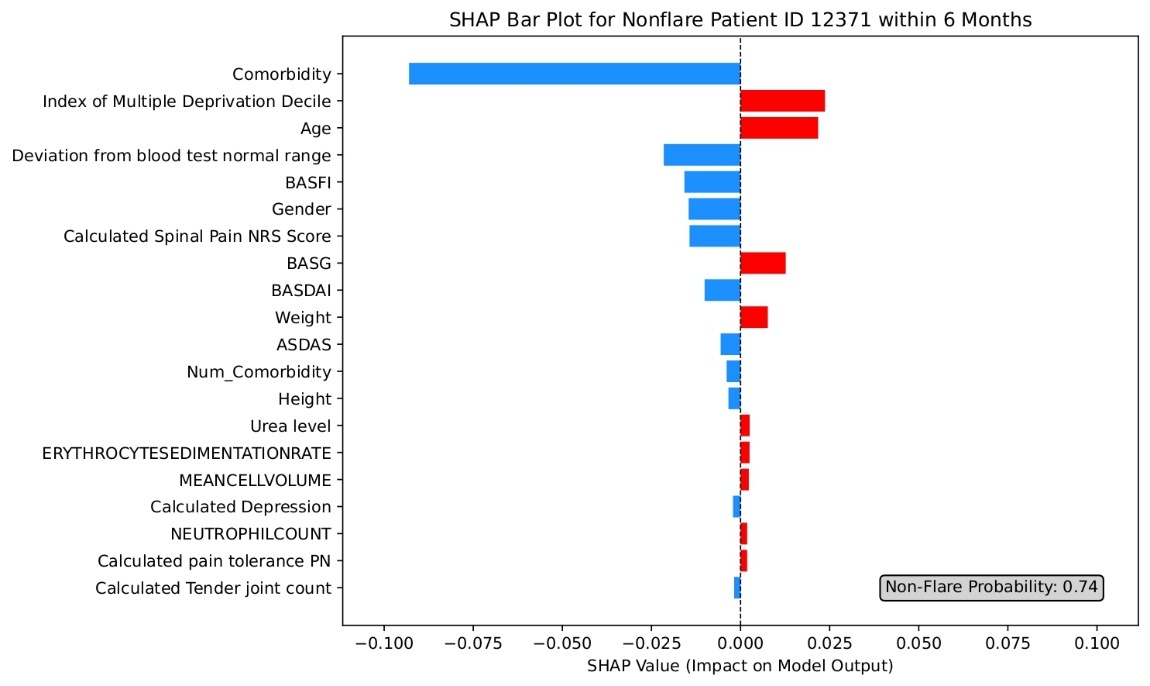


**Figure S27.** The SHAP bar plot presents the main features contributing to the non-flare prediction for Patient ID 12371 within 6 months of next clinic visit with a non-flare likelihood of 0.74. The model's prediction was most affected by Comorbidity, Index of Multiple Deprivation Decile and Age.


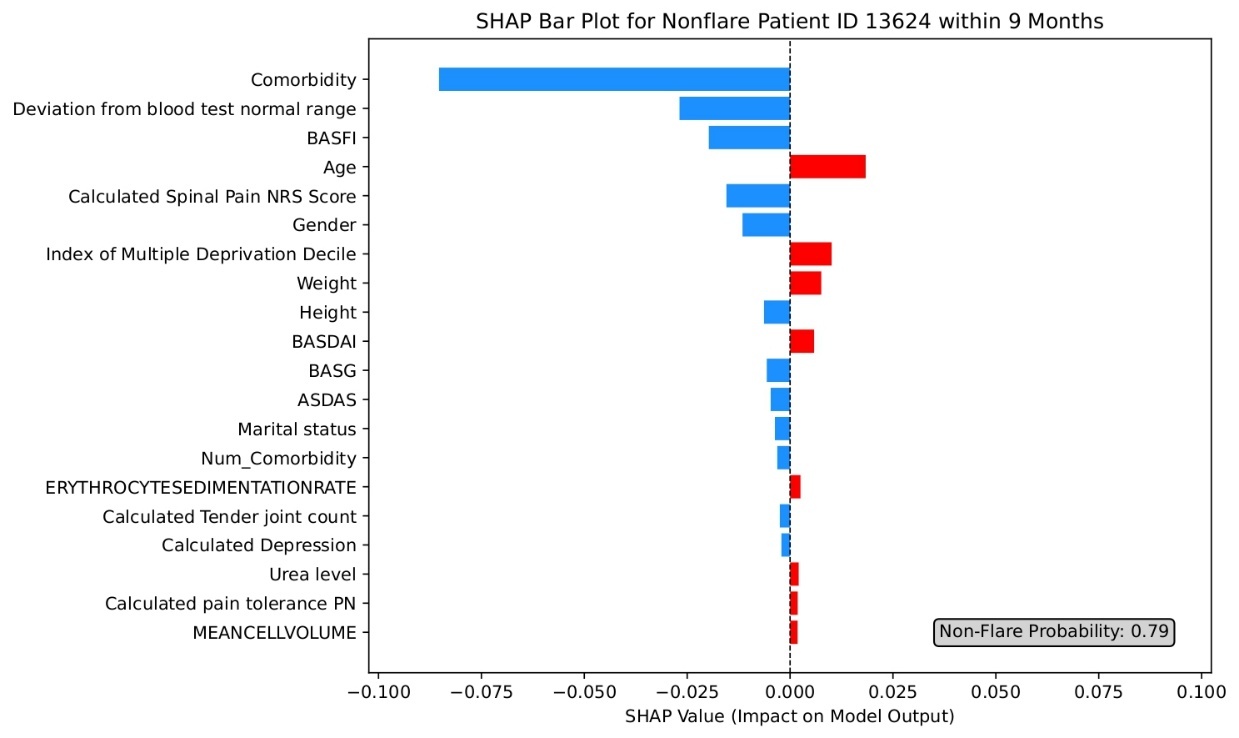


**Figure S28.** The SHAP bar plot shows which features affect the prediction of Patient ID 12371 staying non-flare within 9 months at a 79% probability. Comorbidity, Deviation from blood test normal range, BASFI and Age primarily influenced the prediction.


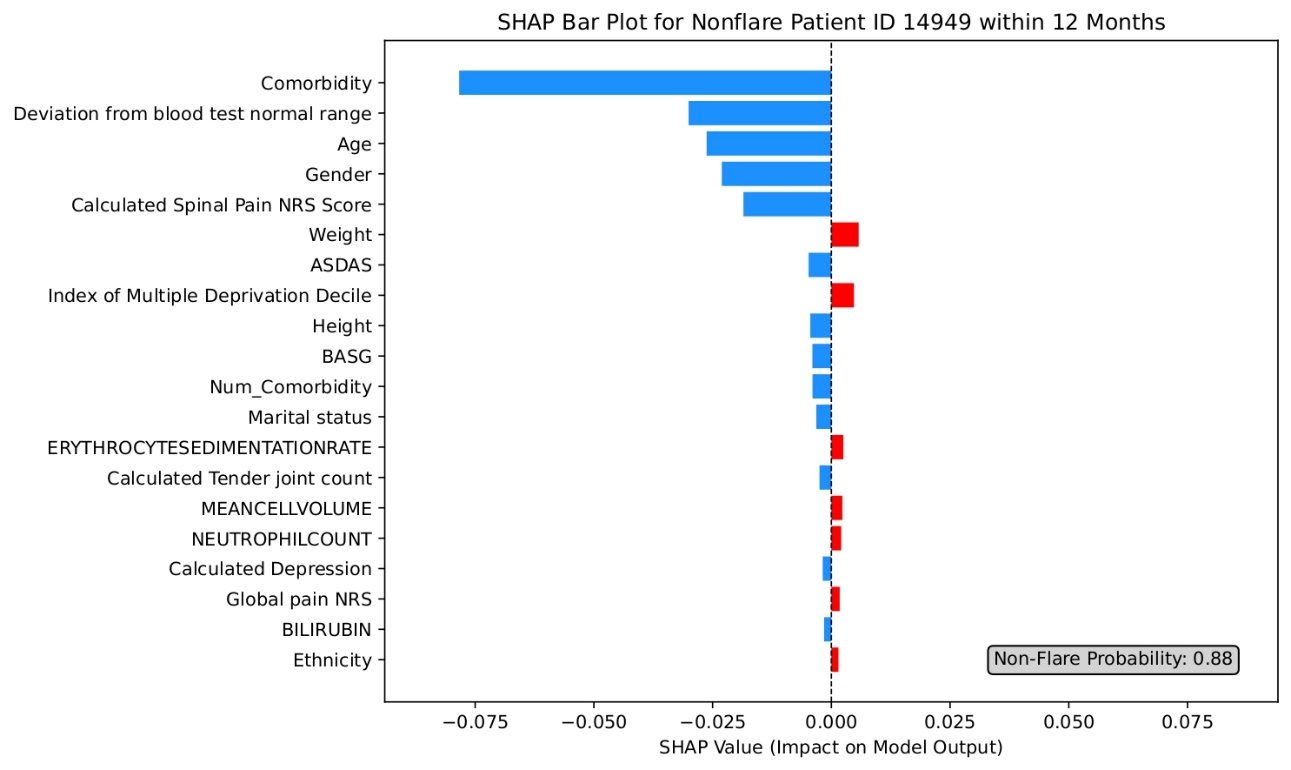


**Figure S29.** The SHAP bar plot shows the main features determining a non-flare outcome prediction for Patient ID 14949 within 12 months with a non-flare probability score of 0.88. Comorbidity, Deviation from blood test normal range, Age, Gender, and Calculated Spinal Pain NRS Score, emerged as significant contributors in the predictive model.

## S12 Supplementary dataset

**SM Dataset S1 (SupplementaryData1.xlsx)**

The Charlson Comorbidity Index (CCI) measurement used ICD-coded diagnoses to calculate comorbidity burden for each patient. Supplementary table presents the comorbidity weights and conditions utilised in the computation process.

**SM Dataset S2 (SupplementaryData2.xlsx)**

The p-values of correlations between clinical features and the Appointment Resource outcome in the testing set are listed in this file. We chose features for further analysis based on their statistically significant associations indicated by p-values below 0.05.

**SM Dataset S3 (SupplementaryData3.xlsx)**

The p-values of correlations between clinical features and the Appointment Resource outcome in the training set are listed in this file. We chose features for further analysis based on their statistically significant associations indicated by p-values below 0.05.

**SM Text S1 (SupplementaryComorbidityCluster.txt)**

The comorbidity list are clusters into different groups.

## S13 Hyperparameter optimisation

**Table S4.** Software packages used in the study, and related hyperparameters search values employed in model selection.

| Method | Software | Hyperparameters search space |
| --- | --- | --- |
| Light Gradient Boosting Tree | Sklearn [3] | colsample_bytree: 0.928  learning_rate: 0.028  max_depth: 9  min_child_samples: 29  n_estimators: 1715  num_leaves: 48  reg_alpha: 0.425  reg_lambda: 7.739  scale_pos_weight: 1.715  subsample: 0.866 |
| Extreme Gradient Boosting | Sklearn [3] | colsample_bytree: 0.801  gamma: 1.712  learning_rate: 0.075  max_depth: 7  min_child_weight: 10  n_estimators: 535  reg_alpha: 1.384  reg_lambda: 1.132  subsample: 0.834 |

## S14 References

[1] Ribeiro, F., and Gradvohl, A.L.S. (2021) 'Machine learning techniques applied to solar flares forecasting', *Astronomy and Computing,*35, pp. 100468.

[2] Cinto, T.*et al.*(2020) 'Solar flare forecasting using time series and extreme gradient boosting ensembles', *Solar Physics,*295(7), pp. 93.

[3] Lars Buitinck, Gilles Louppe, Mathieu Blondel, Fabian Pedregosa, Andreas Mueller, Olivier Grisel, Vlad Niculae, Peter Prettenhofer, Alexandre Gramfort, Jaques Grobler, Robert Layton, Jake VanderPlas, Arnaud Joly, Brian Holt, and Gaël Varoquaux. API design for machine learning software: experiences from the scikit-learn project. In ECML PKDD Workshop: Languages for Data Mining and Machine Learning, pages 108–122, 2013.

[4] Lahmiri, S., Dawson, D.A. and Shmuel, A. (2018) 'Performance of machine learning methods in diagnosing Parkinson’s disease based on dysphonia measures', *Biomedical Engineering Letters,* 8(1), pp. 29–39.
